# Supplementary material for: Objectives and outcomes of patient-driven innovations published in peer-reviewed journals: a qualitative analysis of publications included in a scoping review
Source: BMJ Open. 2023 Jun 1;13(6):e071363. doi: 10.1136/bmjopen-2022-071363 (PMC10255190; doi:10.1136/bmjopen-2022-071363)
Supplement: Supplementary data [file bmjopen-2022-071363supp001.pdf]

Appendix 1. Description of the patient-driven innovations included in the study, grouped by the medical conditions they addressed.

Medical condition	Innovation name	Innovation description	References
22q11 Deletion syndrome	22q11 Ireland	Collaborative network of patients, informal caregivers, clinicians, and researchers for the development a multidisciplinary care coordination and pathways for patients with rare diseases	Lawlor A, Kerin L, Orr D, Leahy R, Crotty F, Kelleher S, et al. Developing integrated care in the context of rare chromosomal conditions: 22q11 Deletion Syndrome; A parent/clinician collaboration. International journal of integrated care. 2017;17(5):215.
Breast cancer	Jacki Jacket	Patient clothing after breast cancer surgery to secure and hide drainage tubes	Berry DL, Blonquist TM, Halpenny B, Hong F, Morrison-Ma SC, McCullough MC, et al. The Jacki Jacket after mastectomy with reconstruction: a randomized pilot study. Breast cancer research and treatment. 2019;179(2):377-85.
Cerebral palsy & hypertonia	Auditory stimulation*	Auditory stimulation to improve motor function in children with cerebral palsy and hypertonia	Ben-Pazi H, Aran A, Pandyan A, Gelkop N, Ginsberg G, Pollak Y, et al. Auditory stimulation improves motor function and caretaker burden in children with cerebral palsy- A randomized double blind study. PLoS One. 2018;13(12):e0208792.
	Upsee	Wearable device. A harness to support gait training for postural control and motor function for children with cerebral palsy and hypertonia	Ardolino E, Flores M, Manella K. Gross Motor Outcomes After Dynamic Weight-Bearing in 2 Children With Trunk Hypotonia: A Case Series. Pediatr Phys Ther. 2017;29(4):360-4.<br><br>Fergus A. A Novel Mobility Device to Improve Walking for a Child With Cerebral Palsy. Pediatr Phys Ther. 2017;29(4):E1-E7.
Cystic fibrosis (CF)	Sweden CF Coalition	Collaborative learning network enabling "persons with CF, families, clinicians, researchers and others to work together toward common goals"	de Monestrol I, Ericson P, Hjelte L, Mostrom J, Hager A, Lindblad S, editors. Co-Creating the Swedish example for orderly introduction and follow-up on new therapies 2018 2018.<br><br>Lindblad A, Hedborg A, Elidottir H, de Monestrol I, Hjelte L, Ericson P, et al., editors. Sweden's learning health system approach to new therapies: nine months with Lumacaftor/Ivacaftor2019 2019.

Diabetes	Autosense	Technical innovation for diabetes care: A development/version of Do-It-Yourself Artificial Pancreas System (DIY-APS), reviewing and assessing insulin sensitivity and adjust ISF deviations	Lewis DM, Leibrand S, Street TJ, Phatak SS. Detecting Insulin Sensitivity Changes for Individuals with Type 1 Diabetes. Diabetes. 2018;67(Supplement_1).
	Autotune	Technical innovation for diabetes care: a development/version of DIY-APS, builds on Nightscout (below) data and recommends changes to basal rate, insulin/carb ratio and ISF	Lewis D. Automatic Estimation of Basals, ISF, and Carb Ratio for Sensor-Augmented Pump and Hybrid Closed-Loop Therapy. American Diabetes Association's 77th Scientific Sessions San Diego, CA2017.
	DIY-APS, Do-It-Yourself Artificial Pancreas System	Digital technical innovation for diabetes care: a closed loop system that enables access to real-time continuous glucose monitoring (CGM) and automated insulin delivery by connecting CGM technology via a communication device to an insulin pump that operates based on an algorithm calculating insulin doses	Barnard KD, Ziegler R, Klonoff DC, Braune K, Petersen B, Rendschmidt T, et al. Open Source Closed-Loop Insulin Delivery Systems: A Clash of Cultures or Merging of Diverse Approaches? Journal of Diabetes Science & Technology. 2018;12(6):1223-6.<br><br>Bock M. The 'do it yourself' type 1 diabetes dilemma for medical practitioners. Internal Medicine Journal. 2019;49(5):559-61.<br><br>Braune K, O'Donnell S, Cleal B, Lewis D, Tappe A, Willaing I, et al. Real-World Use of Do-It-Yourself Artificial Pancreas Systems in Children and Adolescents With Type 1 Diabetes: Online Survey and Analysis of Self-Reported Clinical Outcomes. JMIR mHealth and uHealth. 2019;7(7):e14087-e.<br><br>Burnside M, Crocket H, Mayo M, Pickering J, Tappe A, de Bock M. Do-It-Yourself Automated Insulin Delivery: A Leading Example of the Democratization of Medicine. Journal of diabetes science and technology. 2020;14(5):878-82.<br><br>Crabtree TSJ, McLay A, Wilmot EG. DIY artificial pancreas systems: here to stay? Practical Diabetes. 2019;36(2):63-8.<br><br>Dowling L, Wilmot EG, Choudhary P. Do-it-yourself closed-loop systems for people living with type 1 diabetes. Diabetic Medicine. 2020;37(12):1977-80.

		Farrington C. Hacking diabetes: DIY artificial pancreas systems. Lancet Diabetes Endocrinol. 2017;5(5):332.<br><br>Hng TM, Burren D. Appearance of Do-It-Yourself closed-loop systems to manage type 1 diabetes. Intern Med J. 2018;48(11):1400-4.<br><br>Jennings P, Hussain S. Do-It-Yourself Artificial Pancreas Systems: A Review of the Emerging Evidence and Insights for Healthcare Professionals. J Diabetes Sci Technol. 2020;14(5):868-77.<br><br>Lee JM, Hirschfeld E, Wedding J. A Patient-Designed Do-It-Yourself Mobile Technology System for Diabetes: Promise and Challenges for a New Era in Medicine. JAMA. 2016;315(14):1447-8.<br><br>Lemieux P, Yamamoto J, Donovan L. 44 - Do-It-Yourself Artificial Pancreas System Use in Pregnancy in a Real-World Setting: A Case Report. Canadian Journal of Diabetes. 2020;44(7):S20.<br><br>Lewis D, Leibrand S, Open APSC. Real-World Use of Open Source Artificial Pancreas Systems. J Diabetes Sci Technol. 2016;10(6):1411.<br><br>Lewis D. History and Perspective on DIY Closed Looping. J Diabetes Sci Technol. 2019;13(4):790-3.<br><br>Lewis D. Setting Expectations for Successful Artificial Pancreas/Hybrid Closed Loop/Automated Insulin Delivery Adoption. J Diabetes Sci Technol. 2018;12(2):533-4.<br><br>Lewis DM, Grant AD. 1056-P: Characterization of Multi-timescale Biological Rhythms in Individuals with Type 1 Diabetes. Diabetes. 2019;68 (Supplement_1).<br><br>Lewis DM, Leibrand S. How a DIY Artificial Pancreas Built by a Patient Makes New Tools Available for Clinicians. American Diabetes Association Scientific Sessions 2015.

		Litchman ML, Lewis D, Kelly LA, Gee PM. Twitter Analysis of #OpenAPS DIY Artificial Pancreas Technology Use Suggests Improved A1C and Quality of Life. Journal of diabetes science and technology. 2019;13(2):164-70.<br>Litchman ML, Walker HR, Fitzgerald C, Gomez Hoyos M, Lewis D, Gee PM. Patient-Driven Diabetes Technologies: Sentiment and Personas of the #WeAreNotWaiting and #OpenAPS Movements. Journal of Diabetes Science and Technology. 2020;14(6):990-9.<br><br>Marshall DC, Holloway M, Korer M, Woodman J, Brackenridge A, Hussain S. Do-It-Yourself Artificial Pancreas Systems in Type 1 Diabetes: Perspectives of Two Adult Users, a Caregiver and Three Physicians. Diabetes Ther. 2019;10(5):1553-64.<br><br>Melmer A, Zger T, Lewis DM, Leibrand S, Stettler C, Laimer M. Glycaemic control in individuals with type 1 diabetes using an open source artificial pancreas system (OpenAPS). Diabetes Obesity & Metabolism. 2019;21(10):2333-7.<br><br>Murray JA, Clayton MF, Litchman ML. Health Care Provider Knowledge and Perceptions of FDA-Approved and Do-It-Yourself Automated Insulin Delivery. Journal of diabetes science and technology. 2020;14(6):1017-21.<br><br>O'Donnell S, Lewis D, Marchante Fernández M, Wäldchen M, Cleal B, Skinner T, et al. Evidence on User-Led Innovation in Diabetes Technology (The OPEN Project): Protocol for a Mixed Methods Study. JMIR Research Protocols. 2019;8(11):e15368.<br><br>Oliver N, Reddy M, Marriott C, Walker T, Heinemann L. Open source automated insulin delivery: addressing the challenge. NPJ digital medicine. 2019;2(1):124-.<br><br>Omer T. Empowered citizen 'health hackers' who are not waiting. BMC Medicine. 2016;14(1):118.

		Shaw D, Crabtree TSJ, Hammond P, McLay A, Wilmot EG. The DIY artificial pancreas system: an ethical dilemma for doctors. Diabetic medicine. 2020;37(11):1951-3.<br><br>Shepard JA, Breton M, Nimri R, Roberts JTF, Street T, Klonoff D, et al. User and Healthcare Professional Perspectives on Do-It-Yourself Artificial Pancreas Systems: A Need for Guidelines. Journal of diabetes science and technology. 2022;16(1):224-7.
MySugr	Mobile app, stores diabetes data from connected devices, manual entries, and integrations	Debong F, Mayer H, Kober J. Real-World Assessments of mySugr Mobile Health App. Diabetes Technol Ther. 2019;21(S2):S235-S40.<br><br>Mader JK, Sourij H, Laimer M, Debong F, Schandl B, Pieber TR. Influence of frequeant use of mobile health technology on blood glucose control in patientw with type 1 diabetes 8th International Conference onAdvanced Technologies & Treatments for Diabetes February 18–21, 2015; Paris, France2015.
Nightscout	Technical innovation for diabetes care. Allows remote monitoring of glucose levels of Type 1 diabetes using existing monitoring devices	Årsand E, Bradway M, Muzny M, Blixgård H, Grøttland A, Giordanengo A, et al. Warning: the Do-It-Yourself (DIY) wave will drastically change diabetes care! International Journal of Integrated Care. 2016;16(5):2.<br><br>Beckman D, Reehorst CM, Henriksen A, Muzny M, Arsand E, Hartvigsen G. Better glucose regulation through enabling group-based motivational mechanisms in cloud-based solutions like Nightscout. International Journal of Integrated Care. 2016;16.<br><br>Kublin O, Stępień M. The Nightscout system – description of the system and its evaluation in scientific publications. Pediatric Endocrinology Diabetes and Metabolism. 2020;26(3):140-3.<br><br>Lee JM, Newman MW, Gebremariam A, Choi P, Lewis D, Nordgren W, et al. Real-World Use and Self-Reported Health Outcomes of a Patient-Designed Do-it-Yourself Mobile Technology System for Diabetes: Lessons for Mobile Health. Diabetes Technology & Therapeutics. 2017;19(4):209-19.Journal of diabetes science and technology. 2022;16(1):224-7.

		Ng M, Borst E, Garrity A, Hirschfeld E, Lee J. Evolution of Do-It-Yourself Remote Monitoring Technology for Type 1 Diabetes. Journal of Diabetes Science and Technology. 2020;14(5):854-9.<br><br>Rivard L, Lehoux P, Alami H. "It's not just hacking for the sake of it": a qualitative study of health innovators' views on patient-driven open innovations, quality and safety. BMJ Quality & Safety. 2021;30(9):731-8.<br><br>White K, Gebremariam A, Lewis D, Nordgren W, Wedding J, Pasek J, et al. Motivations for Participation in an Online Social Media Community for Diabetes. Journal of Diabetes Science and Technology. 2018;12(3):712-8.
Omnipod	Technical innovation for diabetes care. A wearable tubeless pump for continuous insulin delivery based on temporary basal rates guided by glucose rise and fall	Lebenthal Y, Lazar L, Benzaquen H, Shalitin S, Phillip M. Patient perceptions of using the OmniPod system compared with conventional insulin pumps in young adults with type 1 diabetes. Diabetes Technol Ther. 2012;14(5):411-7.<br><br>Zisser H, Breton M, Dassau E, Markova K, Bevier W, Seborg D, et al. Novel Methodology to Determine the Accuracy of the OmniPod Insulin Pump: A Key Component of the Artificial Pancreas System. Journal of Diabetes Science and Technology. 2011;5(6):1509-18.
T1resources.uk	Online network. Co-created resource library by healthcare professionals and persons living with Type1 Diabetes to collect and offer valuable and trustworthy self-care resources for diabetes patients	Kendall M, Cleverly L, Winchcombe K, Kar P, Choudhary P, Harris S. T1resources.uk; rated, reviewed, reliable. A co-created website for people affected by type 1 diabetes. British Journal of Diabetes. 2017;17(3).
Webdia	Mobile app for children with Type 1 Diabetes to improve diabetes control by calculating insulin dose and information about meals	Klee P, Bussien C, Castellsague M, Combescure C, Dirlewanger M, Girardin C, et al. An Intervention by a Patient-Designed Do-It-Yourself Mobile Device App Reduces HbA1c in Children and Adolescents with Type 1 Diabetes: A Randomized Double-Crossover Study. Diabetes Technol Ther. 2018;20(12):797-805.

Gastrointestinal diseases	Helminth therapy*	Alternative treatment. Experimental treatment of Crohn's disease by ingesting pig whipworm	Ahrens S. Opening (and Swallowing) A Can of Worms to Treat My Crohn's Disease. Am J Gastroenterol. 2016;111(7):918-20.
	Ostom-i-Alert	Wearable device. An app consisting of a Bluetooth linked stoma bag biosensor that records and alerts patients in real-time to prevent pouch spill	Kontovounisios C, Smith J, Dawson P, Warren O, Mills S, Von Roon A, et al. The Ostom-i Alert Sensor: a new device to measure stoma output. Tech Coloproctol. 2018;22(9):697-701.<br><br>Rouholiman D, Gamble JG, Dobrota SD, Encisco EM, Shah AG, Grajales Iii FJ, et al. Improving Health-Related Quality of Life of Patients With an Ostomy Using a Novel Digital Wearable Device: Protocol for a Pilot Study. JMIR Res Protoc. 2018;7(3):e82.<br><br>Seres M. From Patient to Patient-Entrepreneur: Development of an Ostomy Bag Sensor. Am J Gastroenterol. 2018;113(1):8-10.
Multiple conditions	Genia	App-based patient support system to foster collaborative care to facilitate transition from pediatric to adult care among young chronically ill patients	Grande SW, Longacre MR, Palmblad K, Montan MV, Berquist RP, Hager A, et al. Empowering Young People Living With Juvenile Idiopathic Arthritis to Better Communicate With Families and Care Teams: Content Analysis of Semistructured Interviews. JMIR mHealth and uHealth. 2019;7(2):e10401-e.<br><br>Longacre M, Grande S, Hager A, Montan M, Bergquist RP, Martensson M, et al. Clinical Adoption of mHealth Technology to Support Pediatric Cystic Fibrosis Care in Sweden: Qualitative Case Study. JMIR Pediatr Parent. 2018;1(2):e11080
	MediStori	A paper based personal health record and a self-management toolkit for families	O'Connor O. The MediStori. A personal health record and standardised self-management toolkit which can improve integrated care systems. International Journal of Integrated Care. 2017;17:1-5.
	PatientsLikeMe	Online network empowering patients by enabling sharing of longitudinal health data and information on illness experience (symptoms and treatment and experiences of self-care management through visible profiles	Ma X, Sayama H. Mental disorder recovery correlated with centralities and interactions on an online social network. PeerJ (San Francisco, CA). 2015;3:e1163-e.<br><br>Brownstein CA, Brownstein JS, Williams DS, 3rd, Wicks P, Heywood JA. The power of social networking in medicine. Nat Biotechnol. 2009;27(10):888-90.

		Williams III DS. The PatientsLikeMe® Multiple Sclerosis Community: Using online marketing to shift the health data privacy paradigm. Journal of communication in healthcare. 2010;3(1):48-61.<br><br>Frost J, Massagli M. PatientsLikeMe the case for a data-centered patient community and how ALS patients use the community to inform treatment decisions and manage pulmonary health. Chronic Respiratory Disease. 2009;6(4):225-9.<br><br>Frost JH, Massagli MP. Social uses of personal health information within PatientsLikeMe, an online patient community: what can happen when patients have access to one another's data. J Med Internet Res. 2008;10(3):e15.<br><br>De La Loge C, Dimova S, Mueller K, Phillips G, Durgin TL, Wicks P, et al. PatientsLikeMe® Online Epilepsy Community: Patient characteristics and predictors of poor health-related quality of life. Epilepsy & Behavior. 2016;63:20-8.<br><br>Wicks P, Keininger DL, Massagli MP, de la Loge C, Brownstein C, Isojarvi J, et al. Perceived benefits of sharing health data between people with epilepsy on an online platform. Epilepsy & Behavior. 2012;23(1):16-23.<br><br>Wicks P, Massagli M, Frost J, Brownstein C, Okun S, Vaughan T, et al. Sharing health data for better outcomes on PatientsLikeMe. Journal of Medical Internet Research. 2010;12(2):e19-e.<br><br>Smith CA, Wicks PJ. PatientsLikeMe: Consumer health vocabulary as a folksonomy. AMIA Annual Symposium proceedings. 2008;2008:682-6.<br><br>Sahama T, Liang J, Iannella R. Impact of the social networking applications for health information management for patients and physicians. Studies in health technology and informatics. 2012;180:803-7.

		Frost JH, Massagli MP, Wicks P, Heywood J. How the Social Web Supports patient experimentation with a new therapy: The demand for patient-controlled and patient-centered informatics. AMIA Annual Symposium proceedings. 2008;2008:217-21.<br><br>Griffiths F, Dobermann T, Cave JAK, Thorogood M, Johnson S, Salamatian K, et al. The Impact of Online Social Networks on Health and Health Systems: A Scoping Review and Case Studies. Policy and internet. 2015;7(4):473-96.<br><br>Brownstein CA, Wicks P. The potential research impact of patient reported outcomes on osteogenesis imperfecta. Clin Orthop Relat Res. 2010;468(10):2581-5.<br><br>Wicks P, Heywood JA. Data donation could power the learning health care system, including special access programs. Am J Bioeth. 2014;14(11):27-9.<br><br>Okun S, Goodwin K. Building a learning health community: By the people, for the people. Learning health systems. 2017;1(3):e10028-n/a.<br><br>Chiauzzi E, Wicks P. Digital Trespass: Ethical and Terms-of-Use Violations by Researchers Accessing Data From an Online Patient Community. J Med Internet Res. 2019;21(2):e11985.<br><br>Trevena LJ. PatientsLikeMe and the tale of three brothers. Medical journal of Australia. 2011;195(5):258-9.<br><br>Ellis L, Showell C, Turner P. Social media and patient self-management: not all sites are created equal. Studies in health technology and informatics. 2013;183:291-5.<br><br>Li J. Privacy policies for health social networking sites. Journal of the American Medical Informatics Association : JAMIA. 2013;20(4):704-7.

			Rundle CW, Dellavalle RP. PatientsLikeMe and atopic dermatitis: characterizing the atopic dermatitis patient profile. Dermatology online journal. 2018;24(8).<br>Wicks P. Patient, study thyself. BMC medicine. 2018;16(1):217-.<br><br>Wicks P, Sulham KA, Gnanasakthy A. Quality of Life in Organ Transplant Recipients Participating in an Online Transplant Community. The patient : patient-centered outcomes research. 2013;7(1):73-84.<br><br>O'Brien EC, Rodriguez AM, Kum H-C, Schanberg LE, Fitz-Randolph M, O'Brien SM, et al. Patient perspectives on the linkage of health data for research: Insights from an online patient community questionnaire. International journal of medical informatics (Shannon, Ireland). 2019;127:9-17.
Parkinson's disease	3D cue illusions*	Optical illusion by painting on the floor allowing externally guided steps for patients with Parkinson's disease to alleviate freezing of gait	Janssen S, Soneji M, Nonnekes J, Bloem BR. A painted staircase illusion to alleviate freezing of gait in Parkinson's disease. J Neurol. 2016;263(8):1661-2.<br><br>Janssen S, Wezel RJAv, Soneji M, Nonnekes JH, Bloem BR. Response to: staircase climbing is not solely a visual compensation strategy to alleviate freezing of gait in Parkinson's disease. Journal of neurology. 2017;264(1):177-8.
	Prototype of a mobile app for self-care*	Prototype of a mobile app developed by lead user patients in Parkinson's disease, collecting data on drug intake and well-being for shared decision-making with neurologist	Riggare S, Unruh KT. Patients organise and train doctors to provide better care. BMJ. 2015;351:h6318.
Schizophrenia	Hallucination tracking*	Technical innovation for self-tracking of auditory hallucinations in schizophrenia patients	Torous J, Roux S. Patient-Driven Innovation for Mobile Mental Health Technology: Case Report of Symptom Tracking in Schizophrenia. JMIR Ment Health. 2017;4(3):e27.<br><br>Vaidyam A, Roux S, Torous J. Patient Innovation in Investigating the Effects of Environmental Pollution in Schizophrenia: Case Report of Digital Phenotyping Beyond Apps. JMIR Ment Health. 2020;7(8):e19778.

BMJ Publishing Group Limited (BMJ) disclaims all liability and responsibility arising from any reliance placed on this supplemental material which has been supplied by the author(s)

* A descriptive label is presented for innovations without a name.
