# Supplementary figures and images for: Objectives and outcomes of patient-driven innovations published in peer-reviewed journals: a qualitative analysis of publications included in a scoping review
Source: BMJ Open. 2023 Jun 1;13(6):e071363. doi: 10.1136/bmjopen-2022-071363 (PMC10255190; doi:10.1136/bmjopen-2022-071363)

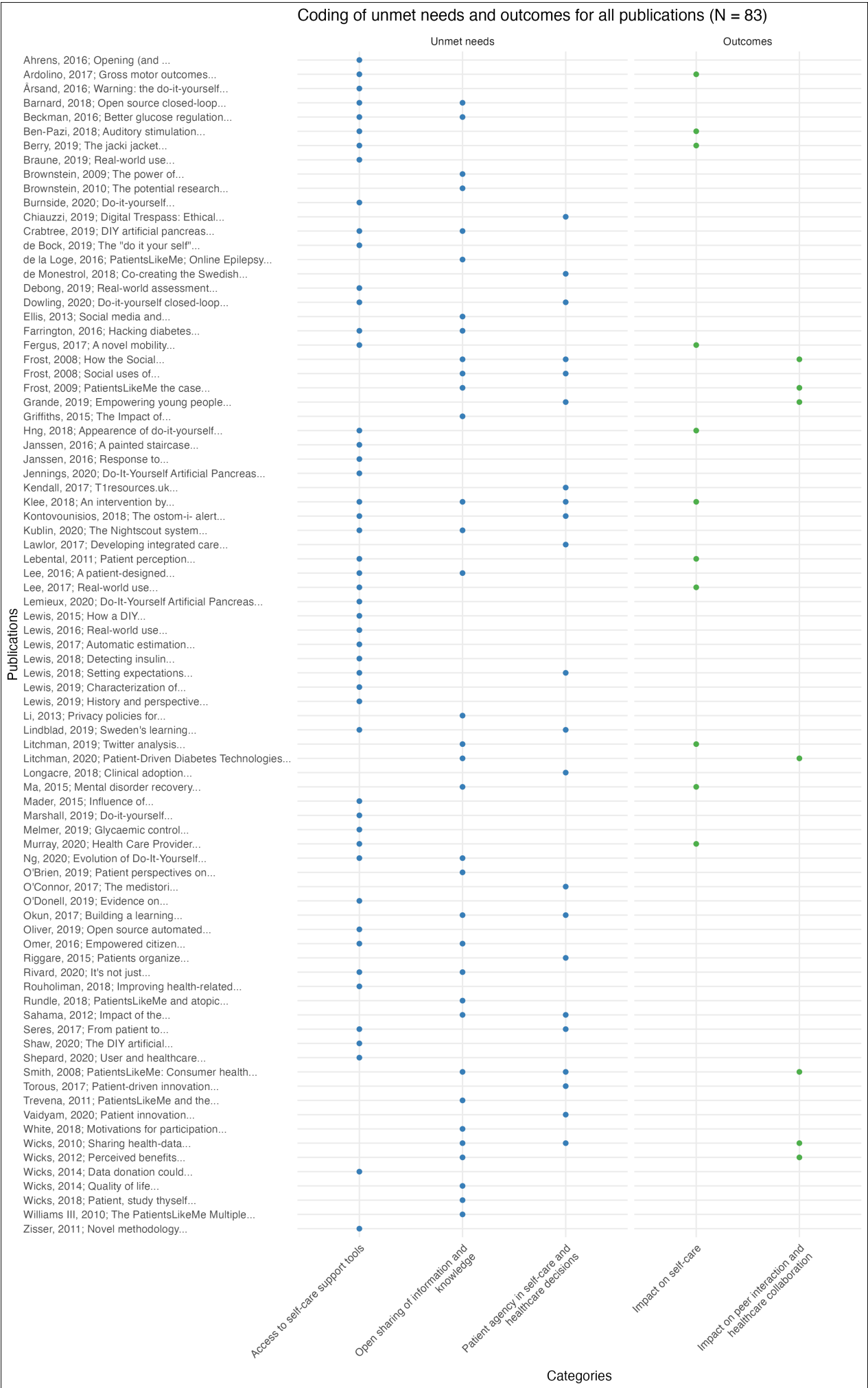

Supplement: Supplementary data [file bmjopen-2022-071363supp002.pdf]
