# Supplementary material for: Objectives and outcomes of patient-driven innovations published in peer-reviewed journals: a qualitative analysis of publications included in a scoping review
Source: BMJ Open. 2023 Jun 1;13(6):e071363. doi: 10.1136/bmjopen-2022-071363 (PMC10255190; doi:10.1136/bmjopen-2022-071363)
Supplement: Author's manuscript [file bmjopen-2022-071363.draft_revisions.pdf]

BMJ Open is committed to open peer review. As part of this commitment we make the peer review history of every article we publish publicly available.

When an article is published we post the peer reviewers' comments and the authors' responses online. We also post the versions of the paper that were used during peer review. These are the versions that the peer review comments apply to.

The versions of the paper that follow are the versions that were submitted during the peer review process. They are not the versions of record or the final published versions. They should not be cited or distributed as the published version of this manuscript.

BMJ Open is an open access journal and the full, final, typeset and author-corrected version of record of the manuscript is available on our site with no access controls, subscription charges or pay-per-view fees (http://bmjopen.bmj.com).

If you have any questions on BMJ Open's open peer review process please email info.bmjopen@bmj.com

BMJ Open

Objectives and outcomes of patient-driven innovations published in peer-reviewed journals: A qualitative analysis of publications included in a scoping review

Journal:	*BMJ Open*
Manuscript ID	bmjopen-2022-071363
Article Type:	Original research
Date Submitted by the Author:	30-Dec-2022

Complete List of Authors:	Dahlberg, Marie; Karolinska Institute Department of Learning Informatics Management and Ethics, ;<br>Lek, Madelen; Karolinska Institute Department of Learning Informatics Management and Ethics<br>Malmqvist, Moa; Karolinska Institute Department of Learning Informatics Management and Ethics<br>Bylund, Ami; Karolinska Institutet, Department of Learning, Informatics, Management and Ethics<br>Hasson, Henna ; Karolinska Institutet, Department of Learning, Informatics, Management and Ethics, Medical Management Centre; Stockholms Lans Landsting, Unit for Implementation and Evaluation, Center for Epidemiology and Community Medicine, Region Stockholm<br>Riggare, Sara; Uppsala Universitet, Participatory eHealth and Health Data, Department of Women's and Children's Health<br>Reinius, Maria; Karolinska Institutet, Department of Learning, Informatics, Management and Ethics<br>Wannheden, Carolina; Karolinska Institutet, Department of Learning, Informatics, Management and Ethics, Medical Management Centre; Karolinska Institutet

Keywords:	Quality in health care < HEALTH SERVICES ADMINISTRATION & MANAGEMENT, PUBLIC HEALTH, QUALITATIVE RESEARCH

SCHOLARONE™
Manuscripts

I, the Submitting Author has the right to grant and does grant on behalf of all authors of the Work (as defined in the below author licence), an exclusive licence and/or a non-exclusive licence for contributions from authors who are: i) UK Crown employees; ii) where BMJ has agreed a CC-BY licence shall apply, and/or iii) in accordance with the terms applicable for US Federal Government officers or employees acting as part of their official duties; on a worldwide, perpetual, irrevocable, royalty-free basis to BMJ Publishing Group Ltd ("BMJ") its licensees and where the relevant Journal is co-owned by BMJ to the co-owners of the Journal, to publish the Work in this journal and any other BMJ products and to exploit all rights, as set out in our licence.

The Submitting Author accepts and understands that any supply made under these terms is made by BMJ to the Submitting Author unless you are acting as an employee on behalf of your employer or a postgraduate student of an affiliated institution which is paying any applicable article publishing charge ("APC") for Open Access articles. Where the Submitting Author wishes to make the Work available on an Open Access basis (and intends to pay the relevant APC), the terms of reuse of such Open Access shall be governed by a Creative Commons licence – details of these licences and which Creative Commons licence will apply to this Work are set out in our licence referred to above.

Other than as permitted in any relevant BMJ Author's Self Archiving Policies, I confirm this Work has not been accepted for publication elsewhere, is not being considered for publication elsewhere and does not duplicate material already published. I confirm all authors consent to publication of this Work and authorise the granting of this licence.

For peer review only

Objectives and outcomes of patient-driven innovations published in peer-reviewed journals: A qualitative analysis of publications included in a scoping review

Marie Dahlberg[1], Madelen Lek[1], Moa Malmqvist Castillo[1], Ami Bylund[1], Henna Hasson[1, 2], Sara Riggare[3], Maria Reinius[1], Carolina Wannheden

Corresponding author:
Marie Dahlberg, Center, Department of Learning, Informatics, Management and Ethics, Medical Management Centre, Karolinska Institutet, Tomtebodavägen 18A, 171 77, Stockholm, Sweden.
E-mail address: marie.dahlberg@ki.se
Phone: +46 (0)705 620268

[1]Department of Learning, Informatics, Management and Ethics, Medical Management Centre, Karolinska Institutet, Stockholm, Sweden

[2]Unit for Implementation and Evaluation, Center for Epidemiology and Community Medicine, Region Stockholm, Stockholm, Sweden

[3]Department of Women's and Children's Health, Healthcare Sciences and E-Health, Uppsala University, Uppsala, Sweden

Key words: patient-driven innovations, self-care, patient agency, peer support, co-creation

Word count: 3818

For peer review only

Abstract

Objectives The objective of this study was gain a deeper understanding of the objectives and outcomes of patient-driven innovations that have been published in the scientific literature, focusing on (a) the unmet needs that patient-driven innovations address, and (b) the outcomes for patients and healthcare that have been reported.

Methods We performed an inductive qualitative content analysis of scientific publications that were included in a scoping review of patient-driven innovations, previously published by our research group. The review was limited to English language publications in peer-reviewed journals, published in the years 2008–2020.

Results In total, 83 publications covering 21 patient-driven innovations were included in the analysis. Most of the innovations were developed for use on an individual or community level without healthcare involvement. We created three categories of unmet needs that were addressed by these innovations: access to self-care support tools, open sharing of information and knowledge, and patient agency in self-care and healthcare decisions. Eighteen (22%) publications reported outcomes of patient-driven innovations. We created two categories of outcomes: impact on self-care, and impact on peer interaction and healthcare collaboration.

Conclusions The patient-driven innovations illustrated a diversity of innovative approaches to facilitate patients' and informal caregivers' daily lives, interactions with peers, and collaborations with healthcare. As our findings indicate, patients and informal caregivers are central stakeholders in driving healthcare development and research forward to meet the needs that matter to patients and informal caregivers. To support wider implementation, more research is needed into regulatory approval processes, dissemination, and governance of patient-driven innovations.

For peer review only

Strengths and limitations in brief

- This study addresses a novel and emerging field of research on patient-driven innovations and builds on a comprehensive scoping review of peer-reviewed literature.
- To our knowledge, this is the first qualitative analysis of published literature on patient-driven innovations, exploring the objectives and outcomes of patient-driven innovations.
- There is a risk of under-representation of research on patient-driven innovations as the drivers of innovations are not always identifiable in scientific publications.
- Given that few publications reported on evaluated outcomes, these findings should be interpreted with some caution.

For peer review only

Background

Multiple policy documents emphasize the importance of involving patients in care decisions and processes (1-3). Also, many patients and their informal caregivers (e.g., parents and family members), increasingly empowered by the improved access to information and technology, expect to actively participate in the decision-making regarding their care (3). Persons living with chronic conditions manage their illness and engage in self-care mainly outside of healthcare. Self-care has been defined as "the ability of individuals, families and communities to promote health, prevent disease, maintain health, and cope with illness and disability with or without the support of a healthcare provider" (4). Self-care practices involve self-care maintenance (i.e., maintaining emotional and physical stability), self-care monitoring (i.e., monitoring changes of symptoms and signs), and self-care management (i.e., managing the symptoms and signs as they occur) (5). In addition to practicing self-care in their daily lives, persons living with chronic conditions are increasingly also engaged in healthcare improvement, where they contribute with their self-care knowledge and experience to improve healthcare processes and services (6, 7). Thus, patients and their informal caregivers are active partners and not only passive recipients of care.

Patients and informal caregivers have developed many innovative solutions to support them in their self-care. For example, an interview study including 500 patients with rare diseases showed that more than half reported developing a solution to improve self-care management of their own diseases (8). The Patient Innovation website, which was created to gather and share innovations developed by patients and informal caregivers, has listed over one thousand solutions (9). These numbers give an indication of significant activity by patients and their

For peer review only

informal caregivers driving health innovations, often independently of the health system. Health innovations have been defined as “new or improved health policies, systems, products and technologies, and services and delivery methods that improve people’s health and wellbeing” (10). In this paper, we use the term patient-driven innovation to describe health innovations that have been initiated and further developed by patients or informal caregivers (11).

A scoping review performed by members in our research team explored the nature and extent of patient-driven innovations published in peer-reviewed journals in the time period from 2008 to 2020; it showed a clearly increasing publication trend of mainly technological innovations in the 2010s (12). As this is a relatively new field of research, the knowledge base about patient-driven innovations reported in peer-reviewed journals is still limited. Therefore, the aim of this study was to gain a deeper understanding of the objectives and outcomes of patient-driven innovations that have been published in peer-reviewed journals, focusing on (a) the unmet needs that patient-driven innovations address, and (b) the outcomes for patients and healthcare that have been reported.

Methods

We reanalyzed the 96 publications that were included in our previously published scoping review by performing an inductive qualitative content analysis (13) of data concerning the objectives and outcomes of patient-driven innovations.

Data collection and analysis

The scoping review was performed by following the framework proposed by Arksey and

For peer review only

O'Malley (14), which has been described in our previous publication (12). Three co-authors (MD, ML, MMC), with supervision from MR, collaborated in qualitatively analyzing the publications. With backgrounds in social and political sciences, bioentrepreneurship, and nursing, they brought several perspectives to the analysis (15). We defined *unmet needs* as the problems or challenges that the patient-driven innovations addressed; if the unmet needs were not explicitly described, we interpreted these based on the descriptions of the patient-driven innovations and their purpose. We defined *outcomes* as any reports of impact of using the patient-driven innovations, including users' self-reported experiences and perceptions. Whereas all publications were analyzed for unmet needs, only publications that were original research studies and were based on empirical data, irrespective of study design, were analyzed for outcomes. First, the researchers collectively read all publications and extracted text corresponding to the content areas of interest (i.e., unmet needs and outcomes) into an Excel spreadsheet; each publication was individually analyzed by two researchers (MD analyzed all publications; ML and MMC each analyzed about half of the publications). The researchers then compared and consolidated their data extractions, whereby uncertainties and conflicts were resolved in discussion. The extracted data constituted the unit of analysis. The analyses were performed collaboratively by MD, ML, and MMC and who created condensed meaning units and labelled these with descriptive codes. The codes were transferred to Miro, an online visual collaboration platform (16). Thereafter, MD, ML, and MMC conducted an iterative process of discussing and refining the codes and grouping them into categories based on similarities in content. Uncertainties regarding the coding and categorization were resolved through discussions among all authors. We report the qualitative findings in combination with descriptive statistics specifying the number and percentage of publications that contributed to

For peer review only

each of the inductively created categories; descriptive statistics are used to provide a more thorough description of our analysis (17). Data about the innovations (e.g., name, description, study design) that were included in the initial review were also used in the analysis to contextualize the findings.

Patient and public involvement

This study was performed within the "Patients in the driver's seat!" research program, where patient innovators and researchers collaborate as partners in research. One of the co-authors is a patient innovator (SR) who contributed to all stages of the research process.

Findings

Of the 96 publications included in the scoping review (12), 13 publications (14%) contained no information that was relevant to the objectives of this study and were therefore excluded. Thus, our analysis included 83 publications, reporting on 21 patient-driven innovations. The publications that were excluded concerned the two innovations (*DIY-APS* [18-24] and *PatientsLikeMe* [25-30]) that accounted for most of the publications included (n = 49, 59%). Therefore, the exclusion of publications did not lead to the exclusion of any patient-driven innovations from the analysis. The characteristics of all innovations, grouped by the medical conditions that they addressed, are described in Appendix 1. The coding of each publication is shown in Appendix 2.

Unmet needs addressed by patient-driven innovations

All the included publications provided information about unmet needs that were drivers for developing an innovation. We created three categories: access to self-care support tools; open

sharing of information and knowledge; and patient agency in self-care and healthcare decisions. Figure 1 illustrates the number of publications represented in each of the categories.

Access to self-care support tools

A central unmet need identified in over half the publications was access to self-care support for physical as well as social activities. The tools and services that patient innovators developed often supported self-care monitoring. For example, a patient innovator used existing technologies to develop the *Ostom-i-Alert* stoma bag sensor that made it easier to monitor stoma output and prevent leakage (31-33). To support continuous treatment follow-up, several innovations comprised mobile applications that made self-monitoring data from various medical devices more easily accessible to patients or their informal caregivers (34-38). In addition to self-care monitoring support, the patient-driven innovations comprised a variety of non-technological products to support physical functioning and participation in social activities. Examples of such self-care management innovations are the *Upsee* harness that supports gait training for children with cerebral palsy (39, 40), and the *Jacki Jacket* that helps breast cancer patients to hide drainage tubes (41). We also found how patient innovators came up with new creative self-care methods that involved no or little product development. For example, the *Auditory stimulation* innovation was an auditory interventional innovation to enhance motor function in children with cerebral palsy; it only required access to a musical sound scheme and a device to play music (42). Similarly, the *3D cue illusions* innovation involved painting a three-dimensional illustration of stairs on the floor to support gait training for people living with Parkinson's disease (43).

For peer review only

Open sharing of information and knowledge

The open sharing of information and knowledge related to self-care was an unmet need addressed in more than one third of the publications. Although most publications concerned the *PatientsLikeMe* platform that enables persons with various health conditions to create personal health profiles, the publications also included innovations with diagnosis-specific platforms (e.g., *DIY-APS, Nightscout,* and *Webdia* for patients with diabetes Type 1). Through these platforms, patients could share their personal health data such as symptoms, treatments, and outcomes within the family (44), or within a wider community of peers (45-47). The platforms thus enabled comparisons of personal health observations with the observations of peers with similar conditions (48).

In addition to platforms for sharing personal health information, patient innovators developed platforms for open sharing of self-care related knowledge. For example, the #WeAreNotWaiting-community hosted a platform for sharing do-it-yourself innovations among patients with diabetes Type 1; patient innovators could openly share code and provide technological assistance to other patients who wanted to build do-it-yourself solutions (49, 50). In this way, do-it-yourself technologies (e.g., *DIY-APS*) could be made available to the broader patient population without requiring regulatory approval (51, 52). Further, this enabled patients to collaborate, contributing to more rapid development and access of innovations.

Patient agency in self-care and healthcare decisions

An unmet need that was addressed by various innovations, covering several different diagnoses, was patient agency in self-care and healthcare decisions. Patient agency is defined as the requirement of "skills across the spectrum of participation in care, ranging from active

For peer review only

participation in medical encounters and decision-making to self-care skills for managing everyday health-related activities" (53). One way to enhance patient agency in self-care was through the curation of trustworthy information and knowledge. Innovations included shared libraries of quality-assured diagnosis-specific self-care information (*T1resources.uk*), as well as personal health records (e.g., *MediStori),* and online health information platforms (e.g., *PatientsLikeMe*) that provided functionalities for collecting, presenting, and comparing personal health data over time, and with others (54-57). These platforms contributed to patient agency in self-care by providing patients with tools to initiate self-monitoring and engaging in self-experimentation, possibly inspired by experiences of other platform users. In addition to tracking personal health data, the *Hallucination tracking* innovation used sensors to collect data from the environment (e.g., air temperature, humidity, air pressure); this enabled hypothesis testing to explore correlations between the occurrence of auditory hallucinations and air quality (55).

Other innovations aimed at improving collaboration with healthcare. For example, mobile patient support systems (e.g., *Genia)* have been developed to support partnership between patients, families, and their care teams by enabling these actors to directly communicate and exchange relevant information (58, 59); in some cases, also enabling integration of patient-reported data into the patients' electronic medical record (33, 59). A way to increase patient agency at higher levels in strategic healthcare decisions was through the establishment of collaborative multi-professional networks, involving patients and family caregivers in the design of care pathways (60), or in the follow-up of therapies (61, 62).

For peer review only

Reported outcomes of patient-driven innovations

Eighteen (22%) publications were original research studies that were based on empirical data and reported outcomes of the patient-driven innovations. Of these, 5 studies (28%) used an experimental study design, 3 (17%) used an observational study design, and 10 (56%) used a descriptive study design. We created two categories representing outcomes: impact on self-care; and impact on peer interaction and healthcare collaboration. Figure 2 illustrates the number of publications represented in each of the categories.

Impact on self-care

Eleven publications reported impacts on self-care, such as improvements in self-care processes, health outcomes, and wellbeing. Several of the innovations for patients with diabetes Type 1 were evaluated with positive outcomes: the *OmniPod* insulin pump was well received by young patients and led to improvements in patients' HbA1c levels (63); *Webdia* also led to a reduction in HbA1c among a subgroup of young patients struggling with their diabetes control but did not affect health-related quality of life compared to usual care (44); and patients and informal caregivers using *DIY-APS* reported an overall reduced burden of illness due to less interference of diabetes with daily life activities (64-66). Although healthcare professionals expressed some uncertainty and concerns regarding such do-it-yourself technologies to their patients, they were open to participating in educational training (67).

Positive impacts on self-care were also reported for innovations that addressed other conditions than diabetes: for example, studies indicated that *auditory stimulation* as well as the *Upsee* harness for gait training led to improved functional motor skills among children

For peer review only

with cerebral palsy (39, 40, 42); breast cancer patients wearing the Jacki Jacket experienced a better body image (41); and engaging in online social interactions using *PatientsLikeMe* was associated with mental recovery among patients with mental disorders (68). None of the publications reported any negative outcomes.

Impact on peer interaction and healthcare collaboration

Seven studies reported impacts on peer interaction and healthcare collaboration. Five of these studies evaluated patients' use and experiences of the *PatientsLikeMe* platform, which contributed mainly to peer interactions. Patients used the platform to ask each other questions, provide advice, and form new relationships (46, 69, 70). Although using the platform was associated with increased comfort in sharing personal health information with others (48), one study highlighted potential challenges in online patient communication due to variation in the terminology that patients use for describing symptoms (71). These findings illustrate how the unmet need for open sharing of information and knowledge among peers was satisfied. Two studies illustrated how patient-driven innovations could also impact collaboration with healthcare, thus strengthening patient agency. The analysis of tweets tagged with #WeAreNotWaiting or #OpenAPS showed that patients as well as healthcare professionals engaged in online conversations about patient-driven diabetes innovations (45). Active and potential users of the patient support system *Genia* experienced or expected that this patient-driven innovation had the potential to improve communication between patients and their healthcare teams through the systematic collection and reporting of patient-reported observations (58).

For peer review only

Discussion

Principal findings

This study investigated 1) the unmet needs that patient-driven innovations published in peer-reviewed journals have addressed and 2) the outcomes that have been reported. We find that the most frequently identified unmet need was availability and access to self-care support, as indicated by the development and spread of self-care innovations aimed at patients and informal caregivers. Reported outcomes suggested that patient-driven innovations could contribute to improved self-care processes, health outcomes, and wellbeing. Other central unmet needs that patient-driven innovations addressed were the ability to learn from others through information and knowledge sharing, and patient agency in self-care and healthcare. Although fewer studies reported outcomes related to these needs, we identified some studies that indicated positive impacts. None of the studies reported any negative outcomes.

Strengths and Limitations

A strength of this study is its contributions to a novel and emerging field of research on patient-driven innovations by building on a comprehensive scoping review of peer-reviewed literature (12). Using the scoping review as a basis for the analysis offered a broad inclusion of articles in the field and ensured a systematic search strategy. Important limitations must also be acknowledged. Due to the chosen inclusion criteria in the scoping review (e.g., English language, publication in peer-reviewed journals), this study may not be representative of all patient-driven innovations that are available. The current findings highlight the unmet needs and outcomes only of innovations that are reported in peer-reviewed journals and could be identified as patient-driven. Future studies could replicate our analysis with other

For peer review only

information sources (e.g., the patient innovation website [9]). Further, the reported outcomes of patient-driven innovations should be interpreted with some caution as they are based on studies with varying designs (experimental, observational, and descriptive) that we did not assess for methodological quality. Our purpose was to gain an understanding of the nature of evaluated and reported outcomes. We excluded studies that were not original research or did not include target end-users (e.g., studies based on expert opinions [72]), which may imply the exclusion of some potentially relevant findings. As no negative outcomes were reported, publication bias cannot be excluded and may even be likely.

Comparison to prior work

Our findings show that patient-driven innovations addressed a variety of unmet needs related to self-care and healthcare, reinforcing earlier studies that highlight the potential of patients and informal caregivers as important drivers of innovation in healthcare (8, 73, 74). By diffusing patient-driven innovations through different channels, including peer-reviewed journals, they have the potential to contribute to unmet needs at a community level. For example, the #WeAreNotWaiting and #OpenAPS movements in diabetes care illustrate how the development and diffusion of patient-driven innovations could radically change the lives of patients, ultimately improving patients' glucose control and reducing caregiver burden (45, 64). The fact that about one third (31%) of the publications included in our analysis concerned the DIY-APS innovation demonstrates how one innovation gained wide attention in the scientific community. This example reflects a culture of open peer-to-peer sharing, speeding up access to innovative solutions (73). However, it should be considered that only a fraction

For peer review only

of all patient-driven innovations is diffused (74). The drivers and barriers to the diffusion and adoption of patient-driven innovations merit further investigation.

Implications for policy and practice

As our findings show, patient-driven innovations have the potential to improve not only self-care processes, but also healthcare processes. However, although some of the publications indicated an interest among healthcare professionals in the opportunities offered by patient-driven innovations, only a few reported outcomes that reflected the use of patient-driven innovations in healthcare. This may be related to existing barriers (e.g., liability, safety, ethics, and policy) to the implementation of patient-driven innovations in healthcare (72, 75, 76). Experts on health innovation and commercialization processes, albeit not necessarily experts on patient-driven innovations, emphasize the importance of clear liability frameworks and governance of patient-driven innovations (72). To address regulatory hurdles, a multidisciplinary approach involving multiple stakeholders (e.g., end-users, healthcare professionals, manufacturers, and regulators) has been recommended (77). A concern is that decision-makers and policymakers often underestimate the value of end-user innovations (75, 76). Thus, to successfully support regulatory approval and implementation of patient-driven innovations in healthcare may require an approach whereby healthcare professionals and researchers recognize and collaborate with patient innovators as independent innovation sources (78). From previous research, there is evidence that co-creation with patients in the design and planning of care can benefit patient-professional relationships, improve health outcomes, and also increase satisfaction among clinicians (79). Further, partnering with patients in research has been associated with improved public health and health-related

For peer review only

outcomes for patients (80). Such co-creation or partnership processes are often driven by researchers or professionals. More efforts may be needed to support and study patient-driven co-creation processes.

Unanswered questions and future research

Although patient-driven innovations are gaining ground, our findings indicate that research on the outcomes of patient-driven innovations is still limited. More evaluation studies will be needed to support the future adoption of patient-driven innovations in various contexts. As such studies require collaboration with researchers and other stakeholders, we could gain valuable insights by exploring the motives and experiences of patient innovators who have participated in such collaborations. Future research should also explore how to build supportive structures for multidisciplinary collaborations that can contribute to quality-assurance, evaluation, and implementation of patient-driven innovations in healthcare.

Conclusions

The patient-driven innovations reported in this study illustrate a diversity of innovative approaches that patients and informal caregivers have applied to address unmet needs related to their self-care, interactions with peers, and collaborations with healthcare. Although positive impacts on self-care, as well as peer interactions, and healthcare collaboration were reported, outcomes of patient-driven innovations need further investigation. To support wider implementation, more research is also needed into regulatory approval processes, dissemination, and governance of patient-driven innovations. This is important because as our findings illustrate, patients and informal caregivers are central stakeholders in driving healthcare development and research forward to meet the needs that truly matter to patients.

For peer review only

Acknowledgements

The authors would like to thank the members in the research program 'Patients in the driver's seat!' for their valuable contributions in the analysis process.

Contributorship statement

All authors contributed to the conceptualization and design of the study and methodology; MD, ML, and MMC extracted and analyzed the data with support from MR and SR; all authors contributed to interpreting the results; MR and HH drafted the introduction; MD drafted the remainder of the manuscript with supervision from CW; all authors revised or critically reviewed the manuscript and read and approved the final draft.

Competing interests

SR who co-authored this paper as a patient innovator and researcher is also an author of one of the publications that was included in the analysis.

Funding

This work was supported by the Swedish Research Council for Health, Working Life and Welfare (FORTE) grant number 2018-01472.

Data sharing statement

All data relevant to this study are included in the article or uploaded as online supplemental information. Online Supplemental files with descriptions of the included patient-driven innovations and coding of unmet needs and outcomes of all publications are available in online supplemental appendices 1 and 2.

For peer review only

Ethical approval

This study does not involve human participants.

For peer review only

References

1. Krist AHAH, Tong STST, Aycock RARA, Longo DRDR. Engaging patients in decision-making and behavior change to promote prevention. Studies in health technology and informatics. 2017;240:284-302.
2. Bombard Y, Baker GR, Orlando E, Fancott C, Bhatia P, Casalino S, et al. Engaging patients to improve quality of care: a systematic review. Implementation Science. 2018;13(1).
3. Meskó B, Debronkart D. Patient Design: The Importance of Including Patients in Designing Health Care. Journal of Medical Internet Research. 2022;24(8):e39178.
4. World Health Organization. What do we mean by self-care? 2020 [Available from: https://www.who.int/reproductivehealth/self-care-interventions/definitions/en/.
5. Riegel B, Jaarsma T, Strömberg A. A middle-range theory of self-care of chronic illness. Advances in nursing science. 2012;35(3):194-204.
6. Granström E, Wannheden C, Brommels M, Hvitfeldt H, Nyström ME. Digital tools as promoters for person-centered care practices in chronic care? Healthcare professionals' experiences from rheumatology care. BMC health services research. 2020;20(1):1108-.
7. Aboumatar H, Pitts S, Sharma R, Das A, Smith BM, Day J, et al. Patient engagement strategies for adults with chronic conditions: an evidence map. Systematic Reviews. 2022;11(1).
8. Oliveira P, Zejnilovic L, Canhao H, von Hippel E. Innovation by patients with rare diseases and chronic needs. Orphanet J Rare Dis. 2015;10:41.
9. Patient Innovation. Patient Innovation - Sharing solutions, Improving life 2022 [Available from: https://patient-innovation.com/.
10. Zafar M, Kristensen F. WHO Health Innovation Group (WHIG). World Health Organization, WHO2016.
11. Oliveira P, Zejnilovic L, Canhão H, von Hippel E. Innovation by patients with rare diseases and chronic needs. Orphanet journal of rare diseases. 2015;10(1):41-.
12. Reinius M, Mazzocato P, Riggare S, Bylund A, Jansson H, Øvretveit J, et al. Patient-driven innovations reported in peer-reviewed journals: a scoping review. BMJ Open. 2022;12(1):e053735.
13. Graneheim UH, Lundman B. Qualitative content analysis in nursing research: concepts, procedures and measures to achieve trustworthiness. Nurse Educ Today. 2004;24(2):105-12.
14. Arksey H, O'Malley L. Scoping studies: towards a methodological framework. International Journal of Social Research Methodology. 2005;8(1):19-32.
15. Cornish F, Gillespie A, Zittoun T. Collaborative Analysis of Qualitative Data, Chaptper published in U. Flick (Éd.), Handbook of qualitative data analysis2013.
16. Miro. Visual collaboration platform [cited 2021 18 November]. Available from: https://miro.com/online-whiteboard/.
17. Sandelowski M. Real qualitative researchers do not count: The use of numbers in qualitative research. Research in Nursing & Health. 2001;24(3):230-40.
18. Braune K, O'Donnell S, Cleal B, Lewis DM, Tappe A, Hauck B, et al. 117-LB: DIWHY: Factors Influencing Motivation, Barriers, and Duration of DIY Artificial Pancreas System Use among Real-World Users. Diabetes (New York, NY). 2019;68(Supplement_1).
19. Cleal B, Braune K, O'Donnell S, Hauck B, Lewis DM, Skinner TC, et al. 78-LB: Detailing the Experiences of People with Diabetes Using Do-It-Yourself Artificial

For peer review only

Pancreas Systems—Qualitative Analysis of Responses to Open-Ended Items in an International Survey. Diabetes (New York, NY). 2019;68(Supplement_1).
20. Hussain S, Lewis D. Diabetes Podcast: Do It Yourself/Open Source Artificial Pancreas Systems: Part 1. Diabetes Therapy. 2020;11(8):1609-19.
21. Hussain S, Lewis D. Diabetes Podcast: Do It Yourself/Open Source Artificial Pancreas Systems: Part 2. Diabetes Therapy. 2020;11(8):1621-6.
22. Lewis DM, Swain RS, Donner TW. Improvements in A1C and Time-in-Range in DIY Closed-Loop (OpenAPS) Users. Diabetes (New York, NY). 2018;67(Supplement 1):352.
23. Zabinsky J, Howell H, Ghezavati A, Lewis DM, Nguyen A, Wong JC. 988-P: Do-It-Yourself Artificial Pancreas Systems for Type 1 Diabetes Reduce Hyperglycemia without Increasing Hypoglycemia. Diabetes. 2020;69(Supplement 1):988-P.
24. Melmer A, Züger T, Lewis D, Leibrand SM, Laimer M. 76-OR: In-Depth Review of Glycemic Control and Glycemic Variability in People with Type 1 Diabetes Using Open Source Artificial Pancreas Systems. Diabetes2019.
25. Heywood J, Zeliadt N. Straight talk with...Jamie Heywood. Nat Med. 2014;20(5):457.
26. Wicks P, Vaughan T, Heywood J. Subjects no more: what happens when trial participants realize they hold the power? BMJ. 2014;348(jan28 9):g368-g.
27. Okun S, Wicks P. DigitalMe: a journey towards personalized health and thriving. Biomedical engineering online. 2018;17(1):119-.
28. Wicks P. Could digital patient communities be the launch pad for patient-centric trial design? Trials. 2014;15.
29. Pearson JF, Brownstein CA, Brownstein JS. Potential for electronic health records and online social networking to redefine medical research. Clinical chemistry (Baltimore, Md). 2011;57(2):196-204.
30. Schroeder EB, Desai J, Schmittdiel JA, Paolino AR, Schneider JL, Goodrich GK, et al. An Innovative Approach to Informing Research: Gathering Perspectives on Diabetes Care Challenges From an Online Patient Community. Interactive journal of medical research. 2015;4(2):e13-e.
31. Rouholiman D, Gamble JG, Dobrota SD, Encisco EM, Shah AG, Grajales Iii FJ, et al. Improving Health-Related Quality of Life of Patients With an Ostomy Using a Novel Digital Wearable Device: Protocol for a Pilot Study. JMIR Res Protoc. 2018;7(3):e82.
32. Seres M. From Patient to Patient-Entrepreneur: Development of an Ostomy Bag Sensor. Am J Gastroenterol. 2018;113(1):8-10.
33. Kontovounisios C, Smith J, Dawson P, Warren O, Mills S, Von Roon A, et al. The Ostom-i Alert Sensor: a new device to measure stoma output. Tech Coloproctol. 2018;22(9):697-701.
34. Lee JM, Hirschfeld E, Wedding J. A Patient-Designed Do-It-Yourself Mobile Technology System for Diabetes: Promise and Challenges for a New Era in Medicine. JAMA. 2016;315(14):1447-8.
35. Kublin O, Stępień M. The Nightscout system – description of the system and its evaluation in scientific publications. Pediatric Endocrinology Diabetes and Metabolism. 2020;26(3):140-3.
36. Ng M, Borst E, Garrity A, Hirschfeld E, Lee J. Evolution of Do-It-Yourself Remote Monitoring Technology for Type 1 Diabetes. Journal of Diabetes Science and Technology. 2020;14(5):854-9.
37. Mader JK, Sourij H, Laimer M, Debong F, Schandl B, Pieber TR. Influence of frequeant use of mobile health technology on blood glucose control in patientw with type 1

For peer review only

diabetes 8th International Conference onAdvanced Technologies & Treatments for Diabetes February 18–21, 2015; Paris, France2015.
38. Debong F, Mayer H, Kober J. Real-World Assessments of mySugr Mobile Health App. Diabetes Technol Ther. 2019;21(S2):S235-S40.
39. Fergus A. A Novel Mobility Device to Improve Walking for a Child With Cerebral Palsy. Pediatr Phys Ther. 2017;29(4):E1-E7.
40. Ardolino E, Flores M, Manella K. Gross Motor Outcomes After Dynamic Weight-Bearing in 2 Children With Trunk Hypotonia: A Case Series. Pediatr Phys Ther. 2017;29(4):360-4.
41. Berry DL, Blonquist TM, Halpenny B, Hong F, Morrison-Ma SC, McCullough MC, et al. The Jacki Jacket after mastectomy with reconstruction: a randomized pilot study. Breast cancer research and treatment. 2019;179(2):377-85.
42. Ben-Pazi H, Aran A, Pandyan A, Gelkop N, Ginsberg G, Pollak Y, et al. Auditory stimulation improves motor function and caretaker burden in children with cerebral palsy- A randomized double blind study. PLoS One. 2018;13(12):e0208792.
43. Janssen S, Soneji M, Nonnekes J, Bloem BR. A painted staircase illusion to alleviate freezing of gait in Parkinson's disease. J Neurol. 2016;263(8):1661-2.
44. Klee P, Bussien C, Castellsague M, Combescure C, Dirlewanger M, Girardin C, et al. An Intervention by a Patient-Designed Do-It-Yourself Mobile Device App Reduces HbA1c in Children and Adolescents with Type 1 Diabetes: A Randomized Double-Crossover Study. Diabetes Technol Ther. 2018;20(12):797-805.
45. Litchman ML, Walker HR, Fitzgerald C, Gomez Hoyos M, Lewis D, Gee PM. Patient-Driven Diabetes Technologies: Sentiment and Personas of the #WeAreNotWaiting and #OpenAPS Movements. Journal of Diabetes Science and Technology. 2020;14(6):990-9.
46. Frost J, Massagli M. PatientsLikeMe the case for a data-centered patient community and how ALS patients use the community to inform treatment decisions and manage pulmonary health. Chronic Respiratory Disease. 2009;6(4):225-9.
47. De La Loge C, Dimova S, Mueller K, Phillips G, Durgin TL, Wicks P, et al. PatientsLikeMe® Online Epilepsy Community: Patient characteristics and predictors of poor health-related quality of life. Epilepsy & Behavior. 2016;63:20-8.
48. Wicks P, Massagli M, Frost J, Brownstein C, Okun S, Vaughan T, et al. Sharing health data for better outcomes on PatientsLikeMe. Journal of Medical Internet Research. 2010;12(2):e19-e.
49. White K, Gebremariam A, Lewis D, Nordgren W, Wedding J, Pasek J, et al. Motivations for Participation in an Online Social Media Community for Diabetes. Journal of Diabetes Science and Technology. 2018;12(3):712-8.
50. Crabtree TSJ, McLay A, Wilmot EG. DIY artificial pancreas systems: here to stay? Practical Diabetes. 2019;36(2):63-8.
51. Barnard KD, Ziegler R, Klonoff DC, Braune K, Petersen B, Rendschmidt T, et al. Open Source Closed-Loop Insulin Delivery Systems: A Clash of Cultures or Merging of Diverse Approaches? Journal of Diabetes Science & Technology. 2018;12(6):1223-6.
52. Farrington C. Hacking diabetes: DIY artificial pancreas systems. Lancet Diabetes Endocrinol. 2017;5(5):332.
53. O'Hair D, Villagran MM, Wittenberg E, Brown K, Ferguson M, Hall HT, et al. Cancer Survivorship and Agency Model: Implications for Patient Choice, Decision Making, and Influence. Health Communication. 2003;15(2):193-202.
54. Frost JH, Massagli MP, Wicks P, Heywood J. How the Social Web Supports patient experimentation with a new therapy: The demand for patient-controlled and patient-centered informatics. AMIA Annual Symposium proceedings. 2008;2008:217-21.

For peer review only

55. Vaidyam A, Roux S, Torous J. Patient Innovation in Investigating the Effects of Environmental Pollution in Schizophrenia: Case Report of Digital Phenotyping Beyond Apps. JMIR Ment Health. 2020;7(8):e19778.
56. O'Connor O. The MediStori. A personal health record and standardised self-management toolkit which can improve integrated care systems. International Journal of Integrated Care. 2017;17:1-5.
57. Kendall M, Cleverly L, Winchcombe K, Kar P, Choudhary P, Harris S. T1resources.uk; rated, reviewed, reliable. A co-created website for people affected by type 1 diabetes. British Journal of Diabetes. 2017;17(3).
58. Grande SW, Longacre MR, Palmblad K, Montan MV, Berquist RP, Hager A, et al. Empowering Young People Living With Juvenile Idiopathic Arthritis to Better Communicate With Families and Care Teams: Content Analysis of Semistructured Interviews. JMIR mHealth and uHealth. 2019;7(2):e10401-e.
59. Longacre M, Grande S, Hager A, Montan M, Bergquist RP, Martensson M, et al. Clinical Adoption of mHealth Technology to Support Pediatric Cystic Fibrosis Care in Sweden: Qualitative Case Study. JMIR Pediatr Parent. 2018;1(2):e11080.
60. Lawlor A, Kerin L, Orr D, Leahy R, Crotty F, Kelleher S, et al. Developing integrated care in the context of rare chromosomal conditions: 22q11 Deletion Syndrome; A parent/clinician collaboration. International journal of integrated care. 2017;17(5):215.
61. de Monestrol I, Ericson P, Hjelte L, Mostrom J, Hager A, Lindblad S, editors. Co-Creating the Swedish example for orderly introduction and follow-up on new therapies 2018 2018.
62. Lindblad A, Hedborg A, Elidottir H, de Monestrol I, Hjelte L, Ericson P, et al., editors. Sweden’s learning health system approach to new therapies: nine months with Lumacaftor/Ivacaftor2019 2019.
63. Lebenthal Y, Lazar L, Benzaquen H, Shalitin S, Phillip M. Patient perceptions of using the OmniPod system compared with conventional insulin pumps in young adults with type 1 diabetes. Diabetes Technol Ther. 2012;14(5):411-7.
64. Hng TM, Burren D. Appearance of Do-It-Yourself closed-loop systems to manage type 1 diabetes. Intern Med J. 2018;48(11):1400-4.
65. Lee JM, Newman MW, Gebremariam A, Choi P, Lewis D, Nordgren W, et al. Real-World Use and Self-Reported Health Outcomes of a Patient-Designed Do-it-Yourself Mobile Technology System for Diabetes: Lessons for Mobile Health. Diabetes Technology & Therapeutics. 2017;19(4):209-19.
66. Litchman ML, Lewis D, Kelly LA, Gee PM. Twitter Analysis of #OpenAPS DIY Artificial Pancreas Technology Use Suggests Improved A1C and Quality of Life. Journal of diabetes science and technology. 2019;13(2):164-70.
67. Murray JA, Clayton MF, Litchman ML. Health Care Provider Knowledge and Perceptions of FDA-Approved and Do-It-Yourself Automated Insulin Delivery. Journal of diabetes science and technology. 2020;14(6):1017-21.
68. Ma X, Sayama H. Mental disorder recovery correlated with centralities and interactions on an online social network. PeerJ (San Francisco, CA). 2015;3:e1163-e.
69. Frost JH, Massagli MP. Social uses of personal health information within PatientsLikeMe, an online patient community: what can happen when patients have access to one another's data. J Med Internet Res. 2008;10(3):e15.
70. Wicks P, Keininger DL, Massagli MP, de la Loge C, Brownstein C, Isojarvi J, et al. Perceived benefits of sharing health data between people with epilepsy on an online platform. Epilepsy & Behavior. 2012;23(1):16-23.
71. Smith CA, Wicks PJ. PatientsLikeMe: Consumer health vocabulary as a folksonomy. AMIA Annual Symposium proceedings. 2008;2008:682-6.

For peer review only

72. Rivard L, Lehoux P, Alami H. "It's not just hacking for the sake of it": a qualitative study of health innovators' views on patient-driven open innovations, quality and safety. BMJ Quality & Safety. 2021;30(9):731-8.
73. Demonaco H, Oliveira P, Torrance A, Von Hippel C, Von Hippel E. When patients become innovators. Springer Fachmedien Wiesbaden; 2020. p. 121-9.
74. Zejnilović L, Oliveira P, Canhão H. Innovations by and for Patients, and Their Place in the Future Health Care System. In: Albach H, Meffert H, Pinkwart A, Reichwald R, von Eiff W, editors. Boundaryless Hospital: Rethink and Redefine Health Care Management. Berlin, Heidelberg: Springer Berlin Heidelberg; 2016. p. 341-57.
75. Bradonjic P, Franke N, Lüthje C. Decision-makers' underestimation of user innovation. Research policy. 2019;48(6):1354-61.
76. Nielsen KR. Policymakers' views on sustainable end-user innovation: Implications for sustainable innovation. Journal of cleaner production. 2020;254:120030.
77. Oliver N, Reddy M, Marriott C, Walker T, Heinemann L. Open source automated insulin delivery: addressing the challenge. NPJ digital medicine. 2019;2(1):124-.
78. Trischler J, Svensson PO, Williams H, Wikström F. Citizens as an innovation source in sustainability transitions - linking the directionality of innovations with the locus of the problem in transformative innovation policy. Public management review. 2022;ahead-of-print(ahead-of-print):1-23.
79. Frow P, McColl-Kennedy JR, Payne A. Co-creation practices: Their role in shaping a health care ecosystem. Industrial Marketing Management. 2016;56:24-39.
80. Halvorsrud K, Kucharska J, Adlington K, Rüdell K, Brown Hajdukova E, Nazroo J, et al. Identifying evidence of effectiveness in the co-creation of research: a systematic review and meta-analysis of the international healthcare literature. Journal of Public Health. 2021;43(1):197-208.

Figure 1: Number and proportion of publications represented in the three inductively created categories representing unmet needs addressed by patient-driven innovations. NB: some publications were included in more than one category.

Figure 2. Number and proportion of publications in the two inductively created categories representing outcomes of patient-driven innovations. NB: in total 65 (78%) of the publications did not report any outcomes.

For peer review only

For peer review only

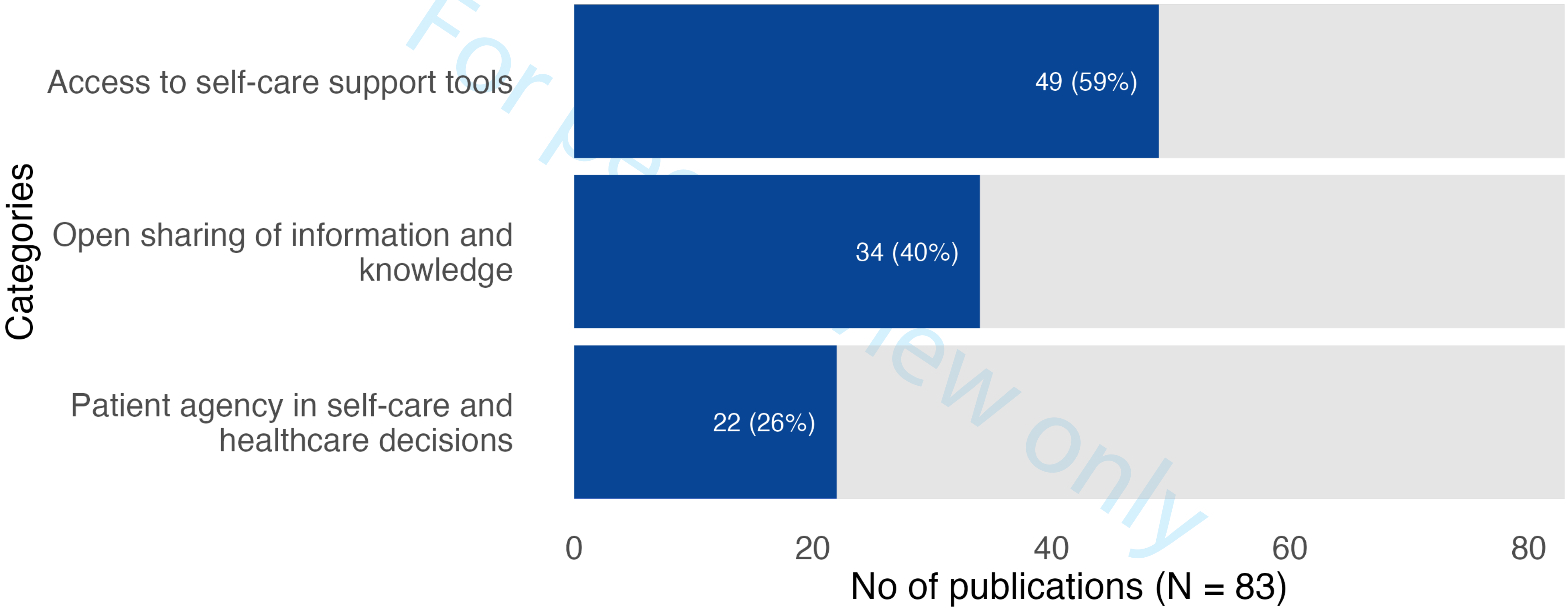

For peer review only - http://bmjopen.bmj.com/site/about/guidelines.xhtml

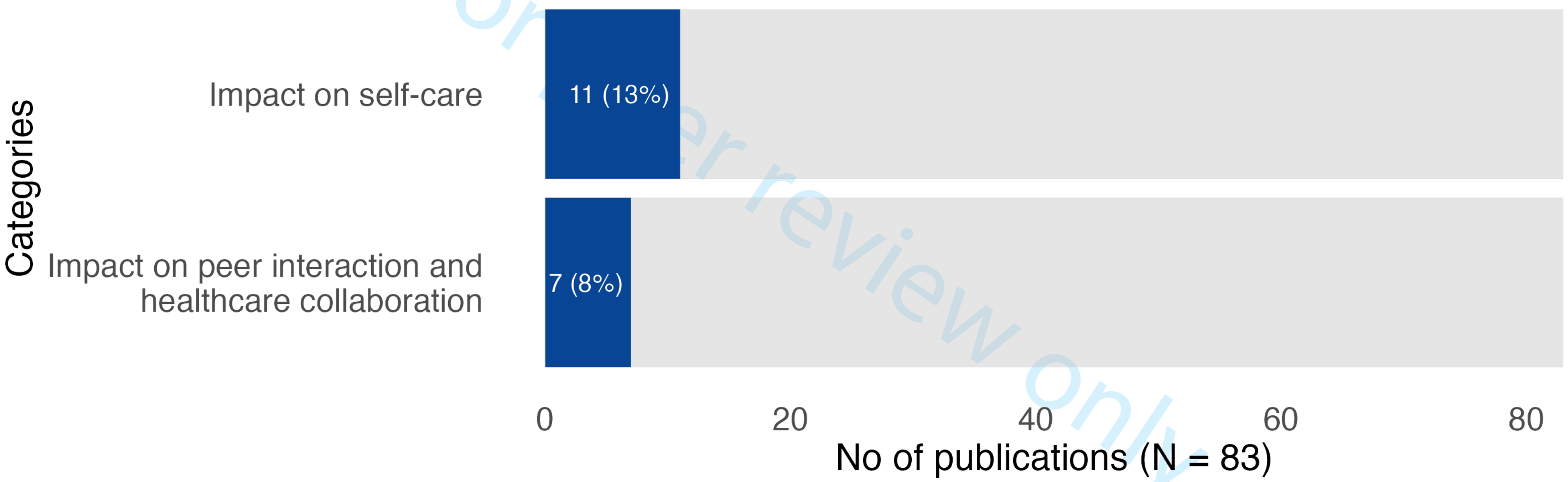

For peer review only

Appendix 1. Description of the patient-driven innovations included in the study, grouped by the medical conditions they addressed.

**Medical condition**	**Innovation name**	**Innovation description**	**References**
22q11 Deletion syndrome	22q11 Ireland	Collaborative network of patients, informal caregivers, clinicians, and researchers for the development a multidisciplinary care coordination and pathways for patients with rare diseases	Lawlor A, Kerin L, Orr D, Leahy R, Crotty F, Kelleher S, et al. Developing integrated care in the context of rare chromosomal conditions: 22q11 Deletion Syndrome; A parent/clinician collaboration. International journal of integrated care. 2017;17(5):215.
Breast cancer	Jacki Jacket	Patient clothing after breast cancer surgery to secure and hide drainage tubes	Berry DL, Blonquist TM, Halpenny B, Hong F, Morrison-Ma SC, McCullough MC, et al. The Jacki Jacket after mastectomy with reconstruction: a randomized pilot study. Breast cancer research and treatment. 2019;179(2):377-85.
Cerebral palsy & hypertonia	Auditory stimulation*	Auditory stimulation to improve motor function in children with cerebral palsy and hypertonia	Ben-Pazi H, Aran A, Pandyan A, Gelkop N, Ginsberg G, Pollak Y, et al. Auditory stimulation improves motor function and caretaker burden in children with cerebral palsy- A randomized double blind study. PLoS One. 2018;13(12):e0208792.
	Upsee	Wearable device. A harness to support gait training for postural control and motor function for children with cerebral palsy and hypertonia	Ardolino E, Flores M, Manella K. Gross Motor Outcomes After Dynamic Weight-Bearing in 2 Children With Trunk Hypotonia: A Case Series. Pediatr Phys Ther. 2017;29(4):360-4.<br><br>Fergus A. A Novel Mobility Device to Improve Walking for a Child With Cerebral Palsy. Pediatr Phys Ther. 2017;29(4):E1-E7.
Cystic fibrosis (CF)	Sweden CF Coalition	Collaborative learning network enabling "persons with CF, families, clinicians, researchers and others to work together toward common goals"	de Monestrol I, Ericson P, Hjelte L, Mostrom J, Hager A, Lindblad S, editors. Co-Creating the Swedish example for orderly introduction and follow-up on new therapies 2018 2018.<br><br>Lindblad A, Hedborg A, Elidottir H, de Monestrol I, Hjelte L, Ericson P, et al., editors. Sweden's learning health system approach to new therapies: nine months with Lumacaftor/Ivacaftor2019 2019.

For peer review only

Diabetes	Autosense	Technical innovation for diabetes care: A development/version of Do-It-Yourself Artificial Pancreas System (DIY-APS), reviewing and assessing insulin sensitivity and adjust ISF deviations	Lewis DM, Leibrand S, Street TJ, Phatak SS. Detecting Insulin Sensitivity Changes for Individuals with Type 1 Diabetes. Diabetes. 2018;67(Supplement_1).
	Autotune	Technical innovation for diabetes care: a development/version of DIY-APS, builds on Nightscout (below) data and recommends changes to basal rate, insulin/carb ratio and ISF	Lewis D. Automatic Estimation of Basals, ISF, and Carb Ratio for Sensor-Augmented Pump and Hybrid Closed-Loop Therapy. American Diabetes Association's 77th Scientific Sessions San Diego, CA2017.
	DIY-APS, Do-It-Yourself Artificial Pancreas System	Digital technical innovation for diabetes care: a closed loop system that enables access to real-time continuous glucose monitoring (CGM) and automated insulin delivery by connecting CGM technology via a communication device to an insulin pump that operates based on an algorithm calculating insulin doses	Barnard KD, Ziegler R, Klonoff DC, Braune K, Petersen B, Rendschmidt T, et al. Open Source Closed-Loop Insulin Delivery Systems: A Clash of Cultures or Merging of Diverse Approaches? Journal of Diabetes Science & Technology. 2018;12(6):1223-6.<br><br>Bock M. The 'do it yourself' type 1 diabetes dilemma for medical practitioners. Internal Medicine Journal. 2019;49(5):559-61.<br><br>Braune K, O'Donnell S, Cleal B, Lewis D, Tappe A, Willaing I, et al. Real-World Use of Do-It-Yourself Artificial Pancreas Systems in Children and Adolescents With Type 1 Diabetes: Online Survey and Analysis of Self-Reported Clinical Outcomes. JMIR mHealth and uHealth. 2019;7(7):e14087-e.<br><br>Burnside M, Crocket H, Mayo M, Pickering J, Tappe A, de Bock M. Do-It-Yourself Automated Insulin Delivery: A Leading Example of the Democratization of Medicine. Journal of diabetes science and technology. 2020;14(5):878-82.<br><br>Crabtree TSJ, McLay A, Wilmot EG. DIY artificial pancreas systems: here to stay? Practical Diabetes. 2019;36(2):63-8.<br><br>Dowling L, Wilmot EG, Choudhary P. Do-it-yourself closed-loop systems for people living with type 1 diabetes. Diabetic Medicine. 2020;37(12):1977-80.

For peer review only

		Farrington C. Hacking diabetes: DIY artificial pancreas systems. Lancet Diabetes Endocrinol. 2017;5(5):332.<br><br>Hng TM, Burren D. Appearance of Do-It-Yourself closed-loop systems to manage type 1 diabetes. Intern Med J. 2018;48(11):1400-4.<br><br>Jennings P, Hussain S. Do-It-Yourself Artificial Pancreas Systems: A Review of the Emerging Evidence and Insights for Healthcare Professionals. J Diabetes Sci Technol. 2020;14(5):868-77.<br><br>Lee JM, Hirschfeld E, Wedding J. A Patient-Designed Do-It-Yourself Mobile Technology System for Diabetes: Promise and Challenges for a New Era in Medicine. JAMA. 2016;315(14):1447-8.<br><br>Lemieux P, Yamamoto J, Donovan L. 44 - Do-It-Yourself Artificial Pancreas System Use in Pregnancy in a Real-World Setting: A Case Report. Canadian Journal of Diabetes. 2020;44(7):S20.<br><br>Lewis D, Leibrand S, Open APSC. Real-World Use of Open Source Artificial Pancreas Systems. J Diabetes Sci Technol. 2016;10(6):1411.<br><br>Lewis D. History and Perspective on DIY Closed Looping. J Diabetes Sci Technol. 2019;13(4):790-3.<br><br>Lewis D. Setting Expectations for Successful Artificial Pancreas/Hybrid Closed Loop/Automated Insulin Delivery Adoption. J Diabetes Sci Technol. 2018;12(2):533-4.<br><br>Lewis DM, Grant AD. 1056-P: Characterization of Multi-timescale Biological Rhythms in Individuals with Type 1 Diabetes. Diabetes. 2019;68 (Supplement_1).<br><br>Lewis DM, Leibrand S. How a DIY Artificial Pancreas Built by a Patient Makes New Tools Available for Clinicians. American Diabetes Association Scientific Sessions 2015.

For peer review only

		Litchman ML, Lewis D, Kelly LA, Gee PM. Twitter Analysis of #OpenAPS DIY Artificial Pancreas Technology Use Suggests Improved A1C and Quality of Life. Journal of diabetes science and technology. 2019;13(2):164-70.<br>Litchman ML, Walker HR, Fitzgerald C, Gomez Hoyos M, Lewis D, Gee PM. Patient-Driven Diabetes Technologies: Sentiment and Personas of the #WeAreNotWaiting and #OpenAPS Movements. Journal of Diabetes Science and Technology. 2020;14(6):990-9.<br><br>Marshall DC, Holloway M, Korer M, Woodman J, Brackenridge A, Hussain S. Do-It-Yourself Artificial Pancreas Systems in Type 1 Diabetes: Perspectives of Two Adult Users, a Caregiver and Three Physicians. Diabetes Ther. 2019;10(5):1553-64.<br><br>Melmer A, Zger T, Lewis DM, Leibrand S, Stettler C, Laimer M. Glycaemic control in individuals with type 1 diabetes using an open source artificial pancreas system (OpenAPS). Diabetes Obesity & Metabolism. 2019;21(10):2333-7.<br><br>Murray JA, Clayton MF, Litchman ML. Health Care Provider Knowledge and Perceptions of FDA-Approved and Do-It-Yourself Automated Insulin Delivery. Journal of diabetes science and technology. 2020;14(6):1017-21.<br><br>O'Donnell S, Lewis D, Marchante Fernández M, Wäldchen M, Cleal B, Skinner T, et al. Evidence on User-Led Innovation in Diabetes Technology (The OPEN Project): Protocol for a Mixed Methods Study. JMIR Research Protocols. 2019;8(11):e15368.<br><br>Oliver N, Reddy M, Marriott C, Walker T, Heinemann L. Open source automated insulin delivery: addressing the challenge. NPJ digital medicine. 2019;2(1):124-.<br><br>Omer T. Empowered citizen 'health hackers' who are not waiting. BMC Medicine. 2016;14(1):118.

For peer review only

		Shaw D, Crabtree TSJ, Hammond P, McLay A, Wilmot EG. The DIY artificial pancreas system: an ethical dilemma for doctors. Diabetic medicine. 2020;37(11):1951-3.<br><br>Shepard JA, Breton M, Nimri R, Roberts JTF, Street T, Klonoff D, et al. User and Healthcare Professional Perspectives on Do-It-Yourself Artificial Pancreas Systems: A Need for Guidelines. Journal of diabetes science and technology. 2022;16(1):224-7.
MySugr	Mobile app, stores diabetes data from connected devices, manual entries, and integrations	Debong F, Mayer H, Kober J. Real-World Assessments of mySugr Mobile Health App. Diabetes Technol Ther. 2019;21(S2):S235-S40.<br><br>Mader JK, Sourij H, Laimer M, Debong F, Schandl B, Pieber TR. Influence of frequeant use of mobile health technology on blood glucose control in patientw with type 1 diabetes 8th International Conference onAdvanced Technologies & Treatments for Diabetes February 18–21, 2015; Paris, France2015.
Nightscout	Technical innovation for diabetes care. Allows remote monitoring of glucose levels of Type 1 diabetes using existing monitoring devices	Årsand E, Bradway M, Muzny M, Blixgård H, Grøttland A, Giordanengo A, et al. Warning: the Do-It-Yourself (DIY) wave will drastically change diabetes care! International Journal of Integrated Care. 2016;16(5):2.<br><br>Beckman D, Reehorst CM, Henriksen A, Muzny M, Arsand E, Hartvigsen G. Better glucose regulation through enabling group-based motivational mechanisms in cloud-based solutions like Nightscout. International Journal of Integrated Care. 2016;16.<br><br>Kublin O, Stępień M. The Nightscout system – description of the system and its evaluation in scientific publications. Pediatric Endocrinology Diabetes and Metabolism. 2020;26(3):140-3.<br><br>Lee JM, Newman MW, Gebremariam A, Choi P, Lewis D, Nordgren W, et al. Real-World Use and Self-Reported Health Outcomes of a Patient-Designed Do-it-Yourself Mobile Technology System for Diabetes: Lessons for Mobile Health. Diabetes Technology & Therapeutics. 2017;19(4):209-19.Journal of diabetes science and technology. 2022;16(1):224-7.

For peer review only

		Ng M, Borst E, Garrity A, Hirschfeld E, Lee J. Evolution of Do-It-Yourself Remote Monitoring Technology for Type 1 Diabetes. Journal of Diabetes Science and Technology. 2020;14(5):854-9.<br><br>Rivard L, Lehoux P, Alami H. "It's not just hacking for the sake of it": a qualitative study of health innovators' views on patient-driven open innovations, quality and safety. BMJ Quality & Safety. 2021;30(9):731-8.<br><br>White K, Gebremariam A, Lewis D, Nordgren W, Wedding J, Pasek J, et al. Motivations for Participation in an Online Social Media Community for Diabetes. Journal of Diabetes Science and Technology. 2018;12(3):712-8.
Omnipod	Technical innovation for diabetes care. A wearable tubeless pump for continuous insulin delivery based on temporary basal rates guided by glucose rise and fall	Lebenthal Y, Lazar L, Benzaquen H, Shalitin S, Phillip M. Patient perceptions of using the OmniPod system compared with conventional insulin pumps in young adults with type 1 diabetes. Diabetes Technol Ther. 2012;14(5):411-7.<br><br>Zisser H, Breton M, Dassau E, Markova K, Bevier W, Seborg D, et al. Novel Methodology to Determine the Accuracy of the OmniPod Insulin Pump: A Key Component of the Artificial Pancreas System. Journal of Diabetes Science and Technology. 2011;5(6):1509-18.
T1resources.uk	Online network. Co-created resource library by healthcare professionals and persons living with Type1 Diabetes to collect and offer valuable and trustworthy self-care resources for diabetes patients	Kendall M, Cleverly L, Winchcombe K, Kar P, Choudhary P, Harris S. T1resources.uk; rated, reviewed, reliable. A co-created website for people affected by type 1 diabetes. British Journal of Diabetes. 2017;17(3).
Webdia	Mobile app for children with Type 1 Diabetes to improve diabetes control by calculating insulin dose and information about meals	Klee P, Bussien C, Castellsague M, Combescure C, Dirlewanger M, Girardin C, et al. An Intervention by a Patient-Designed Do-It-Yourself Mobile Device App Reduces HbA1c in Children and Adolescents with Type 1 Diabetes: A Randomized Double-Crossover Study. Diabetes Technol Ther. 2018;20(12):797-805.

For peer review only

Gastrointestinal diseases	Helminth therapy*	Alternative treatment. Experimental treatment of Crohn's disease by ingesting pig whipworm	Ahrens S. Opening (and Swallowing) A Can of Worms to Treat My Crohn's Disease. Am J Gastroenterol. 2016;111(7):918-20.
	Ostom-i-Alert	Wearable device. An app consisting of a Bluetooth linked stoma bag biosensor that records and alerts patients in real-time to prevent pouch spill	Kontovounisios C, Smith J, Dawson P, Warren O, Mills S, Von Roon A, et al. The Ostom-i Alert Sensor: a new device to measure stoma output. Tech Coloproctol. 2018;22(9):697-701.<br><br>Rouholiman D, Gamble JG, Dobrota SD, Encisco EM, Shah AG, Grajales Iii FJ, et al. Improving Health-Related Quality of Life of Patients With an Ostomy Using a Novel Digital Wearable Device: Protocol for a Pilot Study. JMIR Res Protoc. 2018;7(3):e82.<br><br>Seres M. From Patient to Patient-Entrepreneur: Development of an Ostomy Bag Sensor. Am J Gastroenterol. 2018;113(1):8-10.
Multiple conditions	Genia	App-based patient support system to foster collaborative care to facilitate transition from pediatric to adult care among young chronically ill patients	Grande SW, Longacre MR, Palmblad K, Montan MV, Berquist RP, Hager A, et al. Empowering Young People Living With Juvenile Idiopathic Arthritis to Better Communicate With Families and Care Teams: Content Analysis of Semistructured Interviews. JMIR mHealth and uHealth. 2019;7(2):e10401-e.<br><br>Longacre M, Grande S, Hager A, Montan M, Bergquist RP, Martensson M, et al. Clinical Adoption of mHealth Technology to Support Pediatric Cystic Fibrosis Care in Sweden: Qualitative Case Study. JMIR Pediatr Parent. 2018;1(2):e11080
	MediStori	A paper based personal health record and a self-management toolkit for families	O'Connor O. The MediStori. A personal health record and standardised self-management toolkit which can improve integrated care systems. International Journal of Integrated Care. 2017;17:1-5.
	PatientsLikeMe	Online network empowering patients by enabling sharing of longitudinal health data and information on illness experience (symptoms and treatment and experiences of self-care management through visible profiles	Ma X, Sayama H. Mental disorder recovery correlated with centralities and interactions on an online social network. PeerJ (San Francisco, CA). 2015;3:e1163-e.<br><br>Brownstein CA, Brownstein JS, Williams DS, 3rd, Wicks P, Heywood JA. The power of social networking in medicine. Nat Biotechnol. 2009;27(10):888-90.

For peer review only

		Williams III DS. The PatientsLikeMe® Multiple Sclerosis Community: Using online marketing to shift the health data privacy paradigm. Journal of communication in healthcare. 2010;3(1):48-61.<br><br>Frost J, Massagli M. PatientsLikeMe the case for a data-centered patient community and how ALS patients use the community to inform treatment decisions and manage pulmonary health. Chronic Respiratory Disease. 2009;6(4):225-9.<br><br>Frost JH, Massagli MP. Social uses of personal health information within PatientsLikeMe, an online patient community: what can happen when patients have access to one another's data. J Med Internet Res. 2008;10(3):e15.<br><br>De La Loge C, Dimova S, Mueller K, Phillips G, Durgin TL, Wicks P, et al. PatientsLikeMe® Online Epilepsy Community: Patient characteristics and predictors of poor health-related quality of life. Epilepsy & Behavior. 2016;63:20-8.<br><br>Wicks P, Keininger DL, Massagli MP, de la Loge C, Brownstein C, Isojarvi J, et al. Perceived benefits of sharing health data between people with epilepsy on an online platform. Epilepsy & Behavior. 2012;23(1):16-23.<br><br>Wicks P, Massagli M, Frost J, Brownstein C, Okun S, Vaughan T, et al. Sharing health data for better outcomes on PatientsLikeMe. Journal of Medical Internet Research. 2010;12(2):e19-e.<br><br>Smith CA, Wicks PJ. PatientsLikeMe: Consumer health vocabulary as a folksonomy. AMIA Annual Symposium proceedings. 2008;2008:682-6.<br><br>Sahama T, Liang J, Iannella R. Impact of the social networking applications for health information management for patients and physicians. Studies in health technology and informatics. 2012;180:803-7.

For peer review only

		Frost JH, Massagli MP, Wicks P, Heywood J. How the Social Web Supports patient experimentation with a new therapy: The demand for patient-controlled and patient-centered informatics. AMIA Annual Symposium proceedings. 2008;2008:217-21.<br><br>Griffiths F, Dobermann T, Cave JAK, Thorogood M, Johnson S, Salamatian K, et al. The Impact of Online Social Networks on Health and Health Systems: A Scoping Review and Case Studies. Policy and internet. 2015;7(4):473-96.<br><br>Brownstein CA, Wicks P. The potential research impact of patient reported outcomes on osteogenesis imperfecta. Clin Orthop Relat Res. 2010;468(10):2581-5.<br><br>Wicks P, Heywood JA. Data donation could power the learning health care system, including special access programs. Am J Bioeth. 2014;14(11):27-9.<br><br>Okun S, Goodwin K. Building a learning health community: By the people, for the people. Learning health systems. 2017;1(3):e10028-n/a.<br><br>Chiauzzi E, Wicks P. Digital Trespass: Ethical and Terms-of-Use Violations by Researchers Accessing Data From an Online Patient Community. J Med Internet Res. 2019;21(2):e11985.<br><br>Trevena LJ. PatientsLikeMe and the tale of three brothers. Medical journal of Australia. 2011;195(5):258-9.<br><br>Ellis L, Showell C, Turner P. Social media and patient self-management: not all sites are created equal. Studies in health technology and informatics. 2013;183:291-5.<br><br>Li J. Privacy policies for health social networking sites. Journal of the American Medical Informatics Association : JAMIA. 2013;20(4):704-7.

For peer review only

			Rundle CW, Dellavalle RP. PatientsLikeMe and atopic dermatitis: characterizing the atopic dermatitis patient profile. Dermatology online journal. 2018;24(8).<br>Wicks P. Patient, study thyself. BMC medicine. 2018;16(1):217-.<br><br>Wicks P, Sulham KA, Gnanasakthy A. Quality of Life in Organ Transplant Recipients Participating in an Online Transplant Community. The patient : patient-centered outcomes research. 2013;7(1):73-84.<br><br>O'Brien EC, Rodriguez AM, Kum H-C, Schanberg LE, Fitz-Randolph M, O'Brien SM, et al. Patient perspectives on the linkage of health data for research: Insights from an online patient community questionnaire. International journal of medical informatics (Shannon, Ireland). 2019;127:9-17.
Parkinson's disease	3D cue illusions*	Optical illusion by painting on the floor allowing externally guided steps for patients with Parkinson's disease to alleviate freezing of gait	Janssen S, Soneji M, Nonnekes J, Bloem BR. A painted staircase illusion to alleviate freezing of gait in Parkinson's disease. J Neurol. 2016;263(8):1661-2.<br><br>Janssen S, Wezel RJAv, Soneji M, Nonnekes JH, Bloem BR. Response to: staircase climbing is not solely a visual compensation strategy to alleviate freezing of gait in Parkinson's disease. Journal of neurology. 2017;264(1):177-8.
	Prototype of a mobile app for self-care*	Prototype of a mobile app developed by lead user patients in Parkinson's disease, collecting data on drug intake and well-being for shared decision-making with neurologist	Riggare S, Unruh KT. Patients organise and train doctors to provide better care. BMJ. 2015;351:h6318.
Schizophrenia	Hallucination tracking*	Technical innovation for self-tracking of auditory hallucinations in schizophrenia patients	Torous J, Roux S. Patient-Driven Innovation for Mobile Mental Health Technology: Case Report of Symptom Tracking in Schizophrenia. JMIR Ment Health. 2017;4(3):e27.<br><br>Vaidyam A, Roux S, Torous J. Patient Innovation in Investigating the Effects of Environmental Pollution in Schizophrenia: Case Report of Digital Phenotyping Beyond Apps. JMIR Ment Health. 2020;7(8):e19778.

For peer review only

* A descriptive label is presented for innovations without a name.

For peer review only

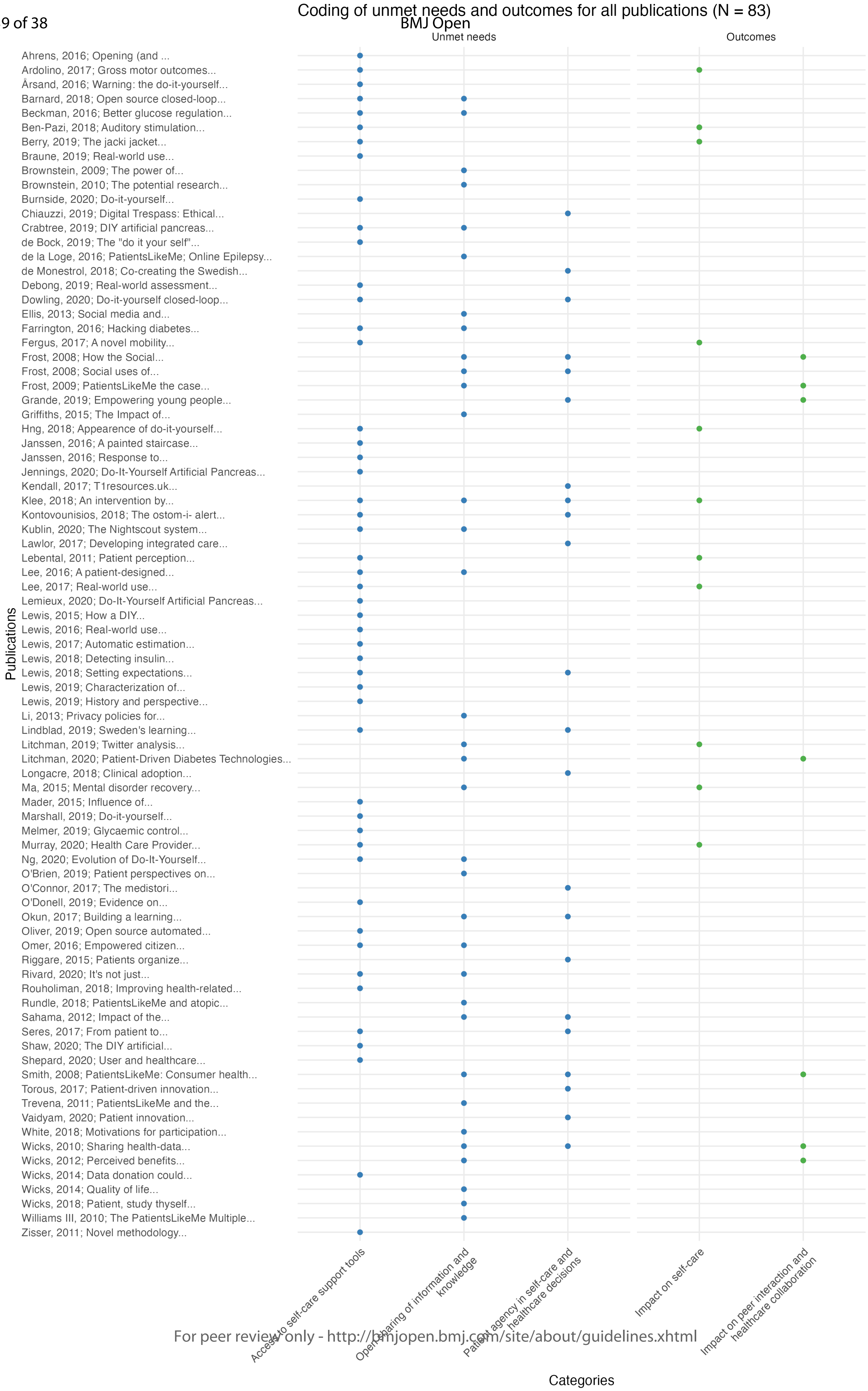
Coding of unmet needs and outcomes for all publications (N = 83)
Unmet needs
Outcomes
Publications
Ahrens, 2016; Opening (and ...
Ardolino, 2017; Gross motor outcomes...
Årsand, 2016; Warning: the do-it-yourself...
Barnard, 2018; Open source closed-loop...
Beckman, 2016; Better glucose regulation...
Ben-Pazi, 2018; Auditory stimulation...
Berry, 2019; The jacki jacket...
Braune, 2019; Real-world use...
Brownstein, 2009; The power of...
Brownstein, 2010; The potential research...
Burnside, 2020; Do-it-yourself...
Chiauzzi, 2019; Digital Trespass: Ethical...
Crabtree, 2019; DIY artificial pancreas...
de Bock, 2019; The "do it your self"...
de la Loge, 2016; PatientsLikeMe; Online Epilepsy...
de Monestrol, 2018; Co-creating the Swedish...
Debong, 2019; Real-world assessment...
Dowling, 2020; Do-it-yourself closed-loop...
Ellis, 2013; Social media and...
Farrington, 2016; Hacking diabetes...
Fergus, 2017; A novel mobility...
Frost, 2008; How the Social...
Frost, 2008; Social uses of...
Frost, 2009; PatientsLikeMe the case...
Grande, 2019; Empowering young people...
Griffiths, 2015; The Impact of...
Hng, 2018; Appearence of do-it-yourself...
Janssen, 2016; A painted staircase...
Janssen, 2016; Response to...
Jennings, 2020; Do-It-Yourself Artificial Pancreas...
Kendall, 2017; T1resources.uk...
Klee, 2018; An intervention by...
Kontovounisios, 2018; The ostom-i- alert...
Kublin, 2020; The Nightscout system...
Lawlor, 2017; Developing integrated care...
Lebental, 2011; Patient perception...
Lee, 2016; A patient-designed...
Lee, 2017; Real-world use...
Lemieux, 2020; Do-It-Yourself Artificial Pancreas...
Lewis, 2015; How a DIY...
Lewis, 2016; Real-world use...
Lewis, 2017; Automatic estimation...
Lewis, 2018; Detecting insulin...
Lewis, 2018; Setting expectations...
Lewis, 2019; Characterization of...
Lewis, 2019; History and perspective...
Li, 2013; Privacy policies for...
Lindblad, 2019; Sweden's learning...
Litchman, 2019; Twitter analysis...
Litchman, 2020; Patient-Driven Diabetes Technologies...
Longacre, 2018; Clinical adoption...
Ma, 2015; Mental disorder recovery...
Mader, 2015; Influence of...
Marshall, 2019; Do-it-yourself...
Melmer, 2019; Glycaemic control...
Murray, 2020; Health Care Provider...
Ng, 2020; Evolution of Do-It-Yourself...
O'Brien, 2019; Patient perspectives on...
O'Connor, 2017; The medistori...
O'Donell, 2019; Evidence on...
Okun, 2017; Building a learning...
Oliver, 2019; Open source automated...
Omer, 2016; Empowered citizen...
Riggare, 2015; Patients organize...
Rivard, 2020; It's not just...
Rouholiman, 2018; Improving health-related...
Rundle, 2018; PatientsLikeMe and atopic...
Sahama, 2012; Impact of the...
Seres, 2017; From patient to...
Shaw, 2020; The DIY artificial...
Shepard, 2020; User and healthcare...
Smith, 2008; PatientsLikeMe: Consumer health...
Torous, 2017; Patient-driven innovation...
Trevena, 2011; PatientsLikeMe and the...
Vaidyam, 2020; Patient innovation...
White, 2018; Motivations for participation...
Wicks, 2010; Sharing health-data...
Wicks, 2012; Perceived benefits...
Wicks, 2014; Data donation could...
Wicks, 2014; Quality of life...
Wicks, 2018; Patient, study thyself...
Williams III, 2010; The PatientsLikeMe Multiple...
Zisser, 2011; Novel methodology...
Access to self-care support tools
Open sharing of information and knowledge
Patient agency in self-care and healthcare decisions
Impact on self-care
Impact on peer interaction and healthcare collaboration
Categories

BMJ Open

Objectives and outcomes of patient-driven innovations published in peer-reviewed journals: A qualitative analysis of publications included in a scoping review

Journal:	*BMJ Open*
Manuscript ID	bmjopen-2022-071363.R1
Article Type:	Original research
Date Submitted by the Author:	10-May-2023
Complete List of Authors:	Dahlberg, Marie; Karolinska Institute Department of Learning Informatics Management and Ethics, ;<br>Lek, Madelen; Karolinska Institute Department of Learning Informatics Management and Ethics<br>Malmqvist, Moa; Karolinska Institute Department of Learning Informatics Management and Ethics<br>Bylund, Ami; Karolinska Institutet, Department of Learning, Informatics, Management and Ethics<br>Hasson, Henna ; Karolinska Institutet, Department of Learning, Informatics, Management and Ethics, Medical Management Centre; Stockholms Lans Landsting, Unit for Implementation and Evaluation, Center for Epidemiology and Community Medicine, Region Stockholm<br>Riggare, Sara; Uppsala Universitet, Participatory eHealth and Health Data, Department of Women's and Children's Health<br>Reinius, Maria; Karolinska Institutet, Department of Learning, Informatics, Management and Ethics<br>Wannheden, Carolina; Karolinska Institutet, Department of Learning, Informatics, Management and Ethics, Medical Management Centre; Karolinska Institutet
**Primary Subject Heading**:	Public health
Secondary Subject Heading:	Medical management
Keywords:	Quality in health care < HEALTH SERVICES ADMINISTRATION & MANAGEMENT, PUBLIC HEALTH, QUALITATIVE RESEARCH

SCHOLARONE™
Manuscripts

I, the Submitting Author has the right to grant and does grant on behalf of all authors of the Work (as defined in the below author licence), an exclusive licence and/or a non-exclusive licence for contributions from authors who are: i) UK Crown employees; ii) where BMJ has agreed a CC-BY licence shall apply, and/or iii) in accordance with the terms applicable for US Federal Government officers or employees acting as part of their official duties; on a worldwide, perpetual, irrevocable, royalty-free basis to BMJ Publishing Group Ltd ("BMJ") its licensees and where the relevant Journal is co-owned by BMJ to the co-owners of the Journal, to publish the Work in this journal and any other BMJ products and to exploit all rights, as set out in our licence.

The Submitting Author accepts and understands that any supply made under these terms is made by BMJ to the Submitting Author unless you are acting as an employee on behalf of your employer or a postgraduate student of an affiliated institution which is paying any applicable article publishing charge ("APC") for Open Access articles. Where the Submitting Author wishes to make the Work available on an Open Access basis (and intends to pay the relevant APC), the terms of reuse of such Open Access shall be governed by a Creative Commons licence – details of these licences and which Creative Commons licence will apply to this Work are set out in our licence referred to above.

Other than as permitted in any relevant BMJ Author's Self Archiving Policies, I confirm this Work has not been accepted for publication elsewhere, is not being considered for publication elsewhere and does not duplicate material already published. I confirm all authors consent to publication of this Work and authorise the granting of this licence.

For peer review only

Objectives and outcomes of patient-driven innovations published in peer-reviewed journals: A qualitative analysis of publications included in a scoping review

Marie Dahlberg[1], Madelen Lek[1], Moa Malmqvist Castillo[1], Ami Bylund[1], Henna Hasson[1, 2], Sara Riggare[3], Maria Reinius[1], Carolina Wannheden[1]

Corresponding author:
Marie Dahlberg, Center, Department of Learning, Informatics, Management and Ethics, Medical Management Centre, Karolinska Institutet, Tomtebodavägen 18A, 171 77, Stockholm, Sweden.
E-mail address: marie.dahlberg@ki.se
Phone: +46 (0)705 620268

[1]Department of Learning, Informatics, Management and Ethics, Medical Management Centre, Karolinska Institutet, Stockholm, Sweden

[2]Unit for Implementation and Evaluation, Center for Epidemiology and Community Medicine, Region Stockholm, Stockholm, Sweden

[3]Department of Women's and Children's Health, Healthcare Sciences and E-Health, Uppsala University, Uppsala, Sweden

Key words: patient-driven innovations, self-care, patient agency, peer support, co-creation

Word count: 3935

For peer review only

ABSTRACT

Objectives The aim of this study was to gain a deeper understanding of the objectives and outcomes of patient-driven innovations that have been published in the scientific literature, focusing on (a) the unmet needs that patient-driven innovations address, and (b) the outcomes for patients and healthcare that have been reported.

Methods We performed an inductive qualitative content analysis of scientific publications that were included in a scoping review of patient-driven innovations, previously published by our research group. The review was limited to English language publications in peer-reviewed journals, published in the years 2008–2020.

Results In total, 83 publications covering 21 patient-driven innovations were included in the analysis. Most of the innovations were developed for use on an individual or community level without healthcare involvement. We created three categories of unmet needs that were addressed by these innovations: access to self-care support tools, open sharing of information and knowledge, and patient agency in self-care and healthcare decisions. Eighteen (22%) publications reported outcomes of patient-driven innovations. We created two categories of outcomes: impact on self-care, and impact on peer interaction and healthcare collaboration.

Conclusions The patient-driven innovations illustrated a diversity of innovative approaches to facilitate patients' and informal caregivers' daily lives, interactions with peers, and collaborations with healthcare. As our findings indicate, patients and informal caregivers are central stakeholders in driving healthcare development and research forward to meet the needs that matter to patients and informal caregivers. However, only few studies reported on outcomes of patient-driven innovations. To support wider implementation, more evaluation studies are needed, as well as research into regulatory approval processes, dissemination, and governance of patient-driven innovations.

For peer review only

STRENGTHS AND LIMITATIONS IN BRIEF

- This study addresses a novel and emerging field of research on patient-driven innovations and builds on a comprehensive scoping review of peer-reviewed literature.
- To our knowledge, this is the first qualitative analysis of published literature on patient-driven innovations, exploring the objectives and outcomes of patient-driven innovations.
- There is a risk of under-representation of research on patient-driven innovations as the drivers of innovations are not always identifiable in scientific publications.
- Given that few publications reported on evaluated outcomes, these findings should be interpreted with some caution.

For peer review only

BACKGROUND

Multiple policy documents emphasize the importance of involving patients in care decisions and processes (1-3). Also, many patients and their informal caregivers (e.g., parents and family members), increasingly empowered by the improved access to information and technology, expect to actively participate in the decision-making regarding their care (3). Active participation may be of particular importance for persons living with chronic conditions as they spend a significant amount of time and energy on managing their illness through self-care. Self-care has been defined as "the ability of individuals, families and communities to promote health, prevent disease, maintain health, and cope with illness and disability with or without the support of a healthcare provider" (4). Self-care practices involve self-care maintenance (i.e., maintaining emotional and physical stability), self-care monitoring (i.e., monitoring changes of symptoms and signs), and self-care management (i.e., managing the symptoms and signs as they occur) (5). In addition to practicing self-care in their daily lives, persons living with chronic conditions are increasingly also engaged in healthcare improvement, where they contribute with their self-care knowledge and experience to improve healthcare processes and services (6, 7). Thus, patients and their informal caregivers are active partners and not only passive recipients of care.

Patients and informal caregivers have developed many innovative solutions to support them in their self-care. For example, an interview study including 500 patients with rare diseases showed that more than half reported developing a solution to improve self-care management of their own diseases (8). The Patient Innovation website, which was created to gather and share innovations developed by patients and informal caregivers, has listed over one thousand solutions (9). These numbers give an indication of significant activity by patients and their

For peer review only - http://bmjopen.bmj.com/site/about/guidelines.xhtml

informal caregivers driving health innovations, often independently of the health system. Health innovations have been defined as "new or improved health policies, systems, products and technologies, and services and delivery methods that improve people's health and wellbeing" (10). In this paper, we use the term patient-driven innovation to describe health innovations that have been initiated and further developed by patients or informal caregivers (11).

A scoping review performed by members in our research team explored the nature and extent of patient-driven innovations published in peer-reviewed journals in the time period from 2008 to 2020; it showed a clearly increasing publication trend of mainly technological innovations in the 2010s; all innovations concerned the management of chronic conditions (11). As this is a relatively new field of research, the knowledge base about patient-driven innovations reported in peer-reviewed journals is still limited. Therefore, the aim of this study was to gain a deeper understanding of the objectives and outcomes of patient-driven innovations that have been published in peer-reviewed journals, focusing on (a) the unmet needs that patient-driven innovations address, and (b) the outcomes for patients and healthcare that have been reported.

METHODS

We reanalyzed the 96 publications that were included in our previously published scoping review by performing an inductive qualitative content analysis (12) of data concerning the objectives and outcomes of patient-driven innovations.

For peer review only

Data collection and analysis

The scoping review was performed by following the framework proposed by Arksey and O'Malley (13), which has been described in our previous publication (11). We inductively analyzed the data by performing a manifest content analysis, which is a suitable method to describe the characteristics of the content by systematically coding the data and staying close to the text, keeping a low level of abstraction throughout the analysis process (12, 14). Three co-authors (MD, ML, MMC), with supervision from MR, collaborated in qualitatively analyzing the publications. With backgrounds in social and political sciences, bioentrepreneurship, and nursing, they brought several perspectives to the analysis (15). We defined *unmet needs* as the problems or challenges that the patient-driven innovations addressed; if the unmet needs were not explicitly described, we interpreted these based on the descriptions of the patient-driven innovations and their purpose. We defined *outcomes* as any reports of impact of using the patient-driven innovations, including users' self-reported experiences and perceptions. Whereas all publications were analyzed for unmet needs, only publications that were original research studies and were based on empirical data, irrespective of study design, were analyzed for outcomes. First, the researchers collectively read all publications and extracted text corresponding to the content areas of interest (i.e., unmet needs and outcomes) into an Excel spreadsheet; each publication was individually analyzed by two researchers (MD analyzed all publications; ML and MMC each analyzed about half of the publications). The researchers then compared and consolidated their data extractions, whereby uncertainties and conflicts were resolved in discussion. The extracted data constituted the unit of analysis. The analyses were performed collaboratively by MD, ML, and MMC and who created condensed meaning units and labelled these with descriptive

For peer review only

codes. The codes were transferred to Miro, an online visual collaboration platform (16). Thereafter, MD, ML, and MMC conducted an iterative process of grouping the codes into categories based on similarities in content. The categorization was discussed among all co-authors and refined until we reached agreement about the level of abstraction and the labelling of the categories. We report the qualitative findings in combination with descriptive statistics specifying the number and percentage of publications that contributed to each of the inductively created categories; descriptive statistics are used to provide a more thorough description of our analysis (17). Data about the innovations (e.g., name, description, study design) that were included in the initial review were also used in the analysis to contextualize the findings.

Patient and public involvement

This study was performed within the "Patients in the driver's seat!" research program, where patient innovators and researchers collaborate as partners in research. One of the co-authors is a patient innovator (SR) who contributed to all stages of the research process.

FINDINGS

Of the 96 publications included in the scoping review (11), 13 publications (14%) contained no information that was relevant to the objectives of this study and were therefore excluded. Thus, our analysis included 83 publications, reporting on 21 patient-driven innovations (18-100). The publications that were excluded concerned the two innovations (*DIY-APS* [101-107] and *PatientsLikeMe* [108-113]) that accounted for most of the publications included (n = 49, 59%). Therefore, the exclusion of publications did not lead to the exclusion of any patient-driven innovations from the analysis. The characteristics of all innovations, grouped by the

For peer review only

medical conditions that they addressed, are described in Appendix 1. The coding of each publication is shown in Appendix 2.

Unmet needs addressed by patient-driven innovations

All the included publications provided information about unmet needs that were drivers for developing an innovation. We created three categories: access to self-care support tools; open sharing of information and knowledge; and patient agency in self-care and healthcare decisions. Figure 1 illustrates the number of publications represented in each of the categories.

Access to self-care support tools

A central unmet need identified in over half the publications was access to self-care support for physical as well as social activities. The tools and services that patient innovators developed often supported self-care monitoring. For example, a patient innovator used existing technologies to develop the *Ostom-i-Alert* stoma bag sensor that made it easier to monitor stoma output and prevent leakage (67-69). To support continuous treatment follow-up, several innovations comprised mobile applications that made self-monitoring data from various medical devices more easily accessible to patients or their informal caregivers (33, 53, 54, 59, 60). In addition to self-care monitoring support, the patient-driven innovations comprised a variety of non-technological products to support physical functioning and participation in social activities. Examples of such self-care management innovations are the *Upsee* harness that supports gait training for children with cerebral palsy (21, 22), and the *Jacki Jacket* that helps breast cancer patients to hide drainage tubes (19). We also found how patient innovators came up with new creative self-care methods that involved no or little product development. For example, the *Auditory stimulation* innovation was an auditory interventional innovation to enhance motor function in children with cerebral palsy; it only

For peer review only

required access to a musical sound scheme and a device to play music (20). Similarly, the *3D cue illusions* innovation involved painting a three-dimensional illustration of stairs on the floor to support gait training for people living with Parkinson's disease (96).

Open sharing of information and knowledge

The open sharing of information and knowledge related to self-care was an unmet need addressed in more than one third of the publications. Although most publications concerned the *PatientsLikeMe* platform that enables persons with various health conditions to create personal health profiles, the publications also included innovations with diagnosis-specific platforms (e.g., *DIY-APS, Nightscout,* and *Webdia* for patients with diabetes Type 1). Through these platforms, patients could share their personal health data such as symptoms, treatments, and outcomes within the family (65), or within a wider community of peers (30, 76, 78). The platforms thus enabled comparisons of personal health observations with the observations of peers with similar conditions (80).

In addition to platforms for sharing personal health information, patient innovators developed platforms for open sharing of self-care related knowledge. For example, the #WeAreNotWaiting-community hosted a platform for sharing do-it-yourself innovations among patients with diabetes Type 1; patient innovators could openly share code and provide technological assistance to other patients who wanted to build do-it-yourself solutions (28, 55). In this way, do-it-yourself technologies (e.g., *DIY-APS*) could be made available to the broader patient population without requiring regulatory approval (36, 39). Further, this enabled patients to collaborate, contributing to more rapid development and access of innovations.

For peer review only

Patient agency in self-care and healthcare decisions

An unmet need that was addressed by various innovations, covering several different diagnoses, was patient agency in self-care and healthcare decisions. Patient agency is defined as the requirement of "skills across the spectrum of participation in care, ranging from active participation in medical encounters and decision-making to self-care skills for managing everyday health-related activities" (114). One way to enhance patient agency in self-care was through the curation of trustworthy information and knowledge. Innovations included shared libraries of quality-assured diagnosis-specific self-care information (*T1resources.uk*), as well as personal health records (e.g., *MediStori),* and online health information platforms (e.g., *PatientsLikeMe*) that provided functionalities for collecting, presenting, and comparing personal health data over time, and with others (64, 72, 83, 99). These platforms contributed to patient agency in self-care by providing patients with tools to initiate self-monitoring and engaging in self-experimentation, possibly inspired by experiences of other platform users. In addition to tracking personal health data, the *Hallucination tracking* innovation used sensors to collect data from the environment (e.g., air temperature, humidity, air pressure); this enabled hypothesis testing to explore correlations between the occurrence of auditory hallucinations and air quality (99).

Other innovations aimed at improving collaboration with healthcare. For example, mobile patient support systems (e.g., *Genia)* have been developed to support partnership between patients, families, and their care teams by enabling these actors to directly communicate and exchange relevant information (70, 71); in some cases, also enabling integration of patient-reported data into the patients' electronic medical record (69, 71). A way to increase patient agency at higher levels in strategic healthcare decisions was through the establishment of

For peer review only

collaborative multi-professional networks, involving patients and family caregivers in the design of care pathways (18), or in the follow-up of therapies (23, 24).

Reported outcomes of patient-driven innovations

Eighteen (22%) publications were original research studies that were based on empirical data and reported outcomes of the patient-driven innovations. Of these, 5 studies (28%) used an experimental study design, 3 (17%) used an observational study design, and 10 (56%) used a descriptive study design. We created two categories representing outcomes: impact on self-care; and impact on peer interaction and healthcare collaboration. Figure 2 illustrates the number of publications represented in each of the categories.

Impact on self-care

Eleven publications reported impacts on self-care, such as improvements in self-care processes, health outcomes, and wellbeing. Several of the innovations for patients with diabetes Type 1 were evaluated with positive outcomes: the *OmniPod* insulin pump was well received by young patients and led to improvements in patients' HbA1c levels (63); *Webdia* also led to a reduction in HbA1c among a subgroup of young patients struggling with their diabetes control but did not affect health-related quality of life compared to usual care (65); and patients and informal caregivers using *DIY-APS* reported an overall reduced burden of illness due to less interference of diabetes with daily life activities (46, 50, 61). Although healthcare professionals expressed some uncertainty and concerns regarding such do-it-yourself technologies to their patients, they were open to participating in educational training (42).

For peer review only

Positive impacts on self-care were also reported for innovations that addressed other conditions than diabetes: for example, studies indicated that *auditory stimulation* as well as the *Upsee* harness for gait training led to improved functional motor skills among children with cerebral palsy (20-22); breast cancer patients wearing the Jacki Jacket experienced a better body image (19); and engaging in online social interactions using *PatientsLikeMe* was associated with mental recovery among patients with mental disorders (73). None of the publications reported any negative outcomes.

Impact on peer interaction and healthcare collaboration

Seven studies reported impacts on peer interaction and healthcare collaboration. Five of these studies evaluated patients' use and experiences of the *PatientsLikeMe* platform, which contributed mainly to peer interactions. Patients used the platform to ask each other questions, provide advice, and form new relationships (76, 77, 79). Although using the platform was associated with increased comfort in sharing personal health information with others (80), one study highlighted potential challenges in online patient communication due to variation in the terminology that patients use for describing symptoms (81). These findings illustrate how the unmet need for open sharing of information and knowledge among peers was satisfied. Two studies illustrated how patient-driven innovations could also impact collaboration with healthcare, thus strengthening patient agency. The analysis of tweets tagged with #WeAreNotWaiting or #OpenAPS showed that patients as well as healthcare professionals engaged in online conversations about patient-driven diabetes innovations (30). Active and potential users of the patient support system *Genia* experienced or expected that this patient-driven innovation had the potential to improve communication between patients and their

For peer review only

healthcare teams through the systematic collection and reporting of patient-reported observations (70).

DISCUSSION

Principal findings

This study investigated 1) the unmet needs that patient-driven innovations published in peer-reviewed journals have addressed and 2) the outcomes that have been reported. We find that the most frequently identified unmet need was availability and access to self-care support, as indicated by the development and spread of self-care innovations aimed at patients and informal caregivers. Reported outcomes suggested that patient-driven innovations could contribute to improved self-care processes, health outcomes, and wellbeing. Other central unmet needs that patient-driven innovations addressed were the ability to learn from others through information and knowledge sharing, and patient agency in self-care and healthcare. Although fewer studies reported outcomes related to these needs, we identified some studies that indicated positive impacts. None of the studies reported any negative outcomes.

Strengths and limitations

A strength of this study is its contributions to a novel and emerging field of research on patient-driven innovations by building on a comprehensive scoping review of peer-reviewed literature (11). Our categorization of unmet needs addressed by patient-driven innovations and reported outcomes may provide a valuable starting point for future research. Using the scoping review as a basis for the analysis offered a broad inclusion of articles in the field and ensured a systematic search strategy. However, important limitations must be acknowledged.

For peer review only

The twenty-one innovations identified in the scoping review are a mere fraction of the patient-driven innovations that have been openly shared through the Patient Innovation platform, which was developed to lower the "cost of sharing" (115, 116). It is likely that the relatively few innovations published in peer-reviewed journals are not a representative sample of all patient-driven innovations that are openly available. The dominance of a few patient-driven innovations (e.g., *PatientsLikeMe* and *DIY-APS*) that accounted for the majority of publications in our sample can probably be explained by a combination of personal characteristics of the innovators, disease characteristics, and opportunities in the respective field. Thus, we emphasize that the current findings highlight the unmet needs and outcomes only of innovations that are reported in peer-reviewed journals and could be identified as patient-driven.

The concept of information power (117), which guides the assessment of sample size in qualitative studies, is also worth considering when interpreting the trustworthiness of our findings. Information power is affected by several factors: for example, a larger sample size may be needed if the aim is broad and the specificity of the sample in relation to the research question is low. The richness of information that was available in the included publications about the unmet needs addressed by the patient-driven innovations and reported outcomes varied. By complementing our data collection with other data sources, such as personal interviews with the innovators, we would probably have been able to gain more information about the objectives and outcomes of the studied innovations and thereby enhance the information power of our sample. Thus, our findings should be interpreted as indicative, giving rise to hypotheses that merit further exploration. In particular, the reported outcomes of

For peer review only

patient-driven innovations should be interpreted with caution as they are based on studies with varying designs (experimental, observational, and descriptive). As no negative outcomes were reported, publication bias cannot be excluded and may even be likely. As common for scoping reviews that address broader topics compared to systematic reviews that may include a quite narrow range of quality assessed studies, we did not assess the methodological quality of included studies (13). Given these limitations, assessing the transferability of our findings to the large number of openly available patient-driven innovations is subject to future research.

Comparison to prior work

Our findings show that patient-driven innovations addressed a variety of unmet needs related to self-care and healthcare, reinforcing earlier studies that highlight the potential of patients and informal caregivers as important drivers of innovation in healthcare (8, 118, 119). By diffusing patient-driven innovations through different channels, including peer-reviewed journals, they have the potential to contribute to unmet needs at a community level. For example, the #WeAreNotWaiting and #OpenAPS movements in diabetes care illustrate how the development and diffusion of patient-driven innovations could radically change the lives of patients, ultimately improving patients' glucose control and reducing caregiver burden (30, 46). The fact that about one third (31%) of the publications included in our analysis concerned the DIY-APS innovation demonstrates how one innovation gained wide attention in the scientific community. This example reflects a culture of open peer-to-peer sharing, speeding up access to innovative solutions (118). However, it should be considered that only a fraction

For peer review only

of all patient-driven innovations is diffused (119). The drivers and barriers to the diffusion and adoption of patient-driven innovations merit further investigation.

Implications for policy and practice

As our findings show, patient-driven innovations have the potential to improve not only self-care processes, but also healthcare processes. However, although some of the publications indicated an interest among healthcare professionals in the opportunities offered by patient-driven innovations, only a few reported outcomes that reflected the use of patient-driven innovations in healthcare. This may be related to existing barriers (e.g., liability, safety, ethics, and policy) to the implementation of patient-driven innovations in healthcare (58, 120, 121). Experts on health innovation and commercialization processes, albeit not necessarily experts on patient-driven innovations, emphasize the importance of clear liability frameworks and governance of patient-driven innovations (58). To address regulatory hurdles, a multidisciplinary approach involving multiple stakeholders (e.g., end-users, healthcare professionals, manufacturers, and regulators) has been recommended (27). A concern is that decision-makers and policymakers often underestimate the value of end-user innovations (120, 121). Thus, to successfully support regulatory approval and implementation of patient-driven innovations in healthcare may require an approach whereby healthcare professionals and researchers recognize and collaborate with patient innovators as independent innovation sources (122). Patient design has been suggested as an approach to engage with patients as true partners in a collaborative process (3). From previous research, there is evidence that co-creation with patients in the design and planning of care can benefit patient-professional relationships, improve health outcomes, and also increase satisfaction among clinicians (123).

For peer review only

Further, partnering with patients in research has been associated with improved public health and health-related outcomes for patients (124). Such co-creation or partnership processes are often driven by researchers or professionals. More efforts may be needed to support and study patient-driven co-creation processes.

Unanswered questions and future research

We call for future studies to explore the transferability of our findings to innovations openly published in the social network space. A recent publication illustrates how machine learning methods can be applied to identify pioneering lead user innovations posted openly on the web that respond to unmet needs, so-called need-solution pairs (125). Such an analysis would likely contribute to developing a more nuanced categorization of unmet needs addressed by patient-driven innovations.

Further, although patient-driven innovations are gaining ground, our findings indicate that research on the outcomes of patient-driven innovations is still limited, which may prevent their adoption in other contexts, such as healthcare. Therefore, more quality evaluation studies may be needed to evaluate the effects of patient-driven innovations. This, in turn, may support the future adoption of effective patient-driven innovations in various contexts. As evaluation studies require collaboration with researchers and possibly other stakeholders, we could gain valuable insights by exploring the motives and experiences of patient innovators who have participated in such collaborations. Patient innovators were co-authors in some, but not all, publications included in our scoping review. Thus, it would be interesting to understand how patient innovators reason about actively engaging in scientific publishing. Future research should also explore how to build supportive structures for multidisciplinary collaborations

For peer review only

that can contribute to quality-assurance, evaluation, and implementation of patient-driven innovations in healthcare.

CONCLUSIONS

The patient-driven innovations reported in this study illustrate a diversity of innovative approaches that patients and informal caregivers have applied to address unmet needs related to their self-care, open sharing of information and knowledge with others, interactions with peers, and collaborations with healthcare. Although positive impacts on self-care, as well as peer interactions, and healthcare collaboration were reported, outcomes of patient-driven innovations need further investigation. To support wider implementation, more research is also needed into regulatory approval processes, dissemination, and governance of patient-driven innovations. This is important because as our findings illustrate, patients and informal caregivers are central stakeholders in driving healthcare development and research forward to meet the needs that truly matter to patients.

ACKNOWLEDGEMENTS

The authors would like to thank the members in the research program 'Patients in the driver's seat!' for their valuable contributions in the analysis process.

CONTRIBUTORSHIP STATEMENT

All authors (MD, ML, MMC, AB, HH, SR, MR, CW) contributed to the conceptualization and design of the study and methodology; MD, ML, and MMC extracted and analyzed the data with support from MR and SR; all authors contributed to interpreting the results; MR and HH drafted the introduction; MD drafted the remainder of the manuscript with

For peer review only

supervision from CW; all authors revised or critically reviewed the manuscript and read and approved the final draft.

COMPETING INTERESTS

SR who co-authored this paper as a patient innovator and researcher is also an author of one of the publications that was included in the analysis.

FUNDING

This work was supported by the Swedish Research Council for Health, Working Life and Welfare / Forskningsrådet om Hälsa, Arbetsliv och Välfärd (FORTE) grant number 2018-01472.

DATA SHARING STATEMENT

All data relevant to this study are included in the article or uploaded as online supplemental information. Online Supplemental files with descriptions of the included patient-driven innovations and coding of unmet needs and outcomes of all publications are available in online supplemental appendices 1 and 2.

ETHICAL APPROVAL

This study does not involve human participants.

REFERENCES

1. Krist AHAH, Tong STST, Aycock RARA, Longo DRDR. Engaging patients in decision-making and behavior change to promote prevention. Studies in health technology and informatics. 2017;240:284-302.

For peer review only

2. Bombard Y, Baker GR, Orlando E, Fancott C, Bhatia P, Casalino S, et al. Engaging patients to improve quality of care: a systematic review. Implementation Science. 2018;13(1).
3. Meskó B, Debronkart D. Patient Design: The Importance of Including Patients in Designing Health Care. Journal of Medical Internet Research. 2022;24(8):e39178.
4. World Health Organization. What do we mean by self-care? 2020 [Available from: https://www.who.int/health-topics/self-care#tab=tab_1.
5. Riegel B, Jaarsma T, Strömberg A. A middle-range theory of self-care of chronic illness. Advances in nursing science. 2012;35(3):194-204.
6. Granström E, Wannheden C, Brommels M, Hvitfeldt H, Nyström ME. Digital tools as promoters for person-centered care practices in chronic care? Healthcare professionals' experiences from rheumatology care. BMC health services research. 2020;20(1):1108-.
7. Aboumatar H, Pitts S, Sharma R, Das A, Smith BM, Day J, et al. Patient engagement strategies for adults with chronic conditions: an evidence map. Systematic Reviews. 2022;11(1).
8. Oliveira P, Zejnilovic L, Canhao H, von Hippel E. Innovation by patients with rare diseases and chronic needs. Orphanet J Rare Dis. 2015;10:41.
9. Patient Innovation. Patient Innovation - Sharing solutions, Improving life 2022 [Available from: https://patient-innovation.com/.
10. Zafar M, Kristensen F. WHO Health Innovation Group (WHIG). World Health Organization, WHO2016.
11. Reinius M, Mazzocato P, Riggare S, Bylund A, Jansson H, Øvretveit J, et al. Patient-driven innovations reported in peer-reviewed journals: a scoping review. BMJ Open. 2022;12(1):e053735.
12. Graneheim UH, Lundman B. Qualitative content analysis in nursing research: concepts, procedures and measures to achieve trustworthiness. Nurse Educ Today. 2004;24(2):105-12.
13. Arksey H, O'Malley L. Scoping studies: towards a methodological framework. International Journal of Social Research Methodology. 2005;8(1):19-32.
14. Graneheim UH, Lindgren B-M, Lundman B. Methodological challenges in qualitative content analysis: A discussion paper. Nurse education today. 2017;56:29-34.
15. Cornish F, Gillespie A, Zittoun T. Collaborative Analysis of Qualitative Data, Chaptper published in U. Flick (Éd.), Handbook of qualitative data analysis2013.
16. Miro. Visual collaboration platform [cited 2021 18 November]. Available from: https://miro.com/online-whiteboard/.
17. Sandelowski M. Real qualitative researchers do not count: The use of numbers in qualitative research. Research in Nursing & Health. 2001;24(3):230-40.
18. Lawlor A, Kerin L, Orr D, Leahy R, Crotty F, Kelleher S, et al. Developing integrated care in the context of rare chromosomal conditions: 22q11 Deletion Syndrome; A parent/clinician collaboration. International journal of integrated care. 2017;17(5):215.
19. Berry DL, Blonquist TM, Halpenny B, Hong F, Morrison-Ma SC, McCullough MC, et al. The Jacki Jacket after mastectomy with reconstruction: a randomized pilot study. Breast cancer research and treatment. 2019;179(2):377-85.
20. Ben-Pazi H, Aran A, Pandyan A, Gelkop N, Ginsberg G, Pollak Y, et al. Auditory stimulation improves motor function and caretaker burden in children with cerebral palsy- A randomized double blind study. PLoS One. 2018;13(12):e0208792.
21. Ardolino E, Flores M, Manella K. Gross Motor Outcomes After Dynamic Weight-Bearing in 2 Children With Trunk Hypotonia: A Case Series. Pediatr Phys Ther. 2017;29(4):360-4.

For peer review only

22. Fergus A. A Novel Mobility Device to Improve Walking for a Child With Cerebral Palsy. Pediatr Phys Ther. 2017;29(4):E1-E7.
23. de Monestrol I, Ericson P, Hjelte L, Mostrom J, Hager A, Lindblad S, editors. Co-Creating the Swedish example for orderly introduction and follow-up on new therapies 2018 2018.
24. Lindblad A, Hedborg A, Elidottir H, de Monestrol I, Hjelte L, Ericson P, et al., editors. Sweden's learning health system approach to new therapies: nine months with Lumacaftor/Ivacaftor2019 2019.
25. Lewis DM, Leibrand S, Street TJ, Phatak SS. Detecting Insulin Sensitivity Changes for Individuals with Type 1 Diabetes. Diabetes. 2018;67(Supplement_1).
26. Lewis D. Automatic Estimation of Basals, ISF, and Carb Ratio for Sensor-Augmented Pump and Hybrid Closed-Loop Therapy. American Diabetes Association's 77th Scientific Sessions San Diego, CA2017.
27. Oliver N, Reddy M, Marriott C, Walker T, Heinemann L. Open source automated insulin delivery: addressing the challenge. NPJ digital medicine. 2019;2(1):124-.
28. Crabtree TSJ, McLay A, Wilmot EG. DIY artificial pancreas systems: here to stay? Practical Diabetes. 2019;36(2):63-8.
29. Lewis D. History and Perspective on DIY Closed Looping. J Diabetes Sci Technol. 2019;13(4):790-3.
30. Litchman ML, Walker HR, Fitzgerald C, Gomez Hoyos M, Lewis D, Gee PM. Patient-Driven Diabetes Technologies: Sentiment and Personas of the #WeAreNotWaiting and #OpenAPS Movements. Journal of Diabetes Science and Technology. 2020;14(6):990-9.
31. Marshall DC, Holloway M, Korer M, Woodman J, Brackenridge A, Hussain S. Do-It-Yourself Artificial Pancreas Systems in Type 1 Diabetes: Perspectives of Two Adult Users, a Caregiver and Three Physicians. Diabetes Ther. 2019;10(5):1553-64.
32. Lewis DM, Leibrand S. How a DIY Artificial Pancreas Built by a Patient Makes New Tools Available for Clinicians. American Diabetes Association Scientific Sessions2015.
33. Lee JM, Hirschfeld E, Wedding J. A Patient-Designed Do-It-Yourself Mobile Technology System for Diabetes: Promise and Challenges for a New Era in Medicine. JAMA. 2016;315(14):1447-8.
34. Lewis D, Leibrand S, Open APSC. Real-World Use of Open Source Artificial Pancreas Systems. J Diabetes Sci Technol. 2016;10(6):1411.
35. Lewis DM, Grant AD. 1056-P: Characterization of Multi-timescale Biological Rhythms in Individuals with Type 1 Diabetes. Diabetes. 2019;68(Supplement_1).
36. Farrington C. Hacking diabetes: DIY artificial pancreas systems. Lancet Diabetes Endocrinol. 2017;5(5):332.
37. Omer T. Empowered citizen 'health hackers' who are not waiting. BMC Medicine. 2016;14(1):118.
38. Shaw D, Crabtree TSJ, Hammond P, McLay A, Wilmot EG. The DIY artificial pancreas system: an ethical dilemma for doctors. Diabetic medicine. 2020;37(11):1951-3.
39. Barnard KD, Ziegler R, Klonoff DC, Braune K, Petersen B, Rendschmidt T, et al. Open Source Closed-Loop Insulin Delivery Systems: A Clash of Cultures or Merging of Diverse Approaches? Journal of Diabetes Science & Technology. 2018;12(6):1223-6.
40. Bock M. The 'do it yourself' type 1 diabetes dilemma for medical practitioners. Internal Medicine Journal. 2019;49(5):559-61.
41. Lewis D. Setting Expectations for Successful Artificial Pancreas/Hybrid Closed Loop/Automated Insulin Delivery Adoption. J Diabetes Sci Technol. 2018;12(2):533-4.
42. Murray JA, Clayton MF, Litchman ML. Health Care Provider Knowledge and Perceptions of FDA-Approved and Do-It-Yourself Automated Insulin Delivery. Journal of diabetes science and technology. 2020;14(6):1017-21.

For peer review only

43. Jennings P, Hussain S. Do-It-Yourself Artificial Pancreas Systems: A Review of the Emerging Evidence and Insights for Healthcare Professionals. J Diabetes Sci Technol. 2020;14(5):868-77.
44. Lemieux P, Yamamoto J, Donovan L. 44 - Do-It-Yourself Artificial Pancreas System Use in Pregnancy in a Real-World Setting: A Case Report. Canadian Journal of Diabetes. 2020;44(7):S20.
45. Dowling L, Wilmot EG, Choudhary P. Do-it-yourself closed-loop systems for people living with type 1 diabetes. Diabetic Medicine. 2020;37(12):1977-80.
46. Hng TM, Burren D. Appearance of Do-It-Yourself closed-loop systems to manage type 1 diabetes. Intern Med J. 2018;48(11):1400-4.
47. Burnside M, Crocket H, Mayo M, Pickering J, Tappe A, de Bock M. Do-It-Yourself Automated Insulin Delivery: A Leading Example of the Democratization of Medicine. Journal of diabetes science and technology. 2020;14(5):878-82.
48. Braune K, O'Donnell S, Cleal B, Lewis D, Tappe A, Willaing I, et al. Real-World Use of Do-It-Yourself Artificial Pancreas Systems in Children and Adolescents With Type 1 Diabetes: Online Survey and Analysis of Self-Reported Clinical Outcomes. JMIR mHealth and uHealth. 2019;7(7):e14087-e.
49. Shepard JA, Breton M, Nimri R, Roberts JTF, Street T, Klonoff D, et al. User and Healthcare Professional Perspectives on Do-It-Yourself Artificial Pancreas Systems: A Need for Guidelines. Journal of diabetes science and technology. 2022;16(1):224-7.
50. Litchman ML, Lewis D, Kelly LA, Gee PM. Twitter Analysis of #OpenAPS DIY Artificial Pancreas Technology Use Suggests Improved A1C and Quality of Life. Journal of diabetes science and technology. 2019;13(2):164-70.
51. O'Donnell S, Lewis D, Marchante Fernández M, Wäldchen M, Cleal B, Skinner T, et al. Evidence on User-Led Innovation in Diabetes Technology (The OPEN Project): Protocol for a Mixed Methods Study. JMIR Research Protocols. 2019;8(11):e15368.
52. Melmer A, Zger T, Lewis DM, Leibrand S, Stettler C, Laimer M. Glycaemic control in individuals with type 1 diabetes using an open source artificial pancreas system (OpenAPS). Diabetes Obesity & Metabolism. 2019;21(10):2333-7.
53. Debong F, Mayer H, Kober J. Real-World Assessments of mySugr Mobile Health App. Diabetes Technol Ther. 2019;21(S2):S235-S40.
54. Mader JK, Sourij H, Laimer M, Debong F, Schandl B, Pieber TR. Influence of frequeant use of mobile health technology on blood glucose control in patientw with type 1 diabetes 8th International Conference onAdvanced Technologies & Treatments for Diabetes February 18–21, 2015; Paris, France2015.
55. White K, Gebremariam A, Lewis D, Nordgren W, Wedding J, Pasek J, et al. Motivations for Participation in an Online Social Media Community for Diabetes. Journal of Diabetes Science and Technology. 2018;12(3):712-8.
56. Årsand E, Bradway M, Muzny M, Blixgård H, Grøttland A, Giordanengo A, et al. Warning: the Do-It-Yourself (DIY) wave will drastically change diabetes care! International Journal of Integrated Care. 2016;16(5):2.
57. Beckman D, Reehorst CM, Henriksen A, Muzny M, Arsand E, Hartvigsen G. Better glucose regulation through enabling group-based motivational mechanisms in cloud-based solutions like Nightscout. International Journal of Integrated Care. 2016;16.
58. Rivard L, Lehoux P, Alami H. "It's not just hacking for the sake of it": a qualitative study of health innovators' views on patient-driven open innovations, quality and safety. BMJ Quality & Safety. 2021;30(9):731-8.
59. Kublin O, Stępień M. The Nightscout system – description of the system and its evaluation in scientific publications. Pediatric Endocrinology Diabetes and Metabolism. 2020;26(3):140-3.

For peer review only

60. Ng M, Borst E, Garrity A, Hirschfeld E, Lee J. Evolution of Do-It-Yourself Remote Monitoring Technology for Type 1 Diabetes. Journal of Diabetes Science and Technology. 2020;14(5):854-9.
61. Lee JM, Newman MW, Gebremariam A, Choi P, Lewis D, Nordgren W, et al. Real-World Use and Self-Reported Health Outcomes of a Patient-Designed Do-it-Yourself Mobile Technology System for Diabetes: Lessons for Mobile Health. Diabetes Technology & Therapeutics. 2017;19(4):209-19.
62. Zisser H, Breton M, Dassau E, Markova K, Bevier W, Seborg D, et al. Novel Methodology to Determine the Accuracy of the OmniPod Insulin Pump: A Key Component of the Artificial Pancreas System. Journal of Diabetes Science and Technology. 2011;5(6):1509-18.
63. Lebenthal Y, Lazar L, Benzaquen H, Shalitin S, Phillip M. Patient perceptions of using the OmniPod system compared with conventional insulin pumps in young adults with type 1 diabetes. Diabetes Technol Ther. 2012;14(5):411-7.
64. Kendall M, Cleverly L, Winchcombe K, Kar P, Choudhary P, Harris S. T1resources.uk; rated, reviewed, reliable. A co-created website for people affected by type 1 diabetes. British Journal of Diabetes. 2017;17(3).
65. Klee P, Bussien C, Castellsague M, Combescure C, Dirlewanger M, Girardin C, et al. An Intervention by a Patient-Designed Do-It-Yourself Mobile Device App Reduces HbA1c in Children and Adolescents with Type 1 Diabetes: A Randomized Double-Crossover Study. Diabetes Technol Ther. 2018;20(12):797-805.
66. Ahrens S. Opening (and Swallowing) A Can of Worms to Treat My Crohn's Disease. Am J Gastroenterol. 2016;111(7):918-20.
67. Rouholiman D, Gamble JG, Dobrota SD, Encisco EM, Shah AG, Grajales Iii FJ, et al. Improving Health-Related Quality of Life of Patients With an Ostomy Using a Novel Digital Wearable Device: Protocol for a Pilot Study. JMIR Res Protoc. 2018;7(3):e82.
68. Seres M. From Patient to Patient-Entrepreneur: Development of an Ostomy Bag Sensor. Am J Gastroenterol. 2018;113(1):8-10.
69. Kontovounisios C, Smith J, Dawson P, Warren O, Mills S, Von Roon A, et al. The Ostom-i Alert Sensor: a new device to measure stoma output. Tech Coloproctol. 2018;22(9):697-701.
70. Grande SW, Longacre MR, Palmblad K, Montan MV, Berquist RP, Hager A, et al. Empowering Young People Living With Juvenile Idiopathic Arthritis to Better Communicate With Families and Care Teams: Content Analysis of Semistructured Interviews. JMIR mHealth and uHealth. 2019;7(2):e10401-e.
71. Longacre M, Grande S, Hager A, Montan M, Bergquist RP, Martensson M, et al. Clinical Adoption of mHealth Technology to Support Pediatric Cystic Fibrosis Care in Sweden: Qualitative Case Study. JMIR Pediatr Parent. 2018;1(2):e11080.
72. O'Connor O. The MediStori. A personal health record and standardised self-management toolkit which can improve integrated care systems. International Journal of Integrated Care. 2017;17:1-5.
73. Ma X, Sayama H. Mental disorder recovery correlated with centralities and interactions on an online social network. PeerJ (San Francisco, CA). 2015;3:e1163-e.
74. Brownstein CA, Brownstein JS, Williams DS, 3rd, Wicks P, Heywood JA. The power of social networking in medicine. Nat Biotechnol. 2009;27(10):888-90.
75. Williams III DS. The PatientsLikeMe® Multiple Sclerosis Community: Using online marketing to shift the health data privacy paradigm. Journal of communication in healthcare. 2010;3(1):48-61.

For peer review only

76. Frost J, Massagli M. PatientsLikeMe the case for a data-centered patient community and how ALS patients use the community to inform treatment decisions and manage pulmonary health. Chronic Respiratory Disease. 2009;6(4):225-9.
77. Frost JH, Massagli MP. Social uses of personal health information within PatientsLikeMe, an online patient community: what can happen when patients have access to one another's data. J Med Internet Res. 2008;10(3):e15.
78. De La Loge C, Dimova S, Mueller K, Phillips G, Durgin TL, Wicks P, et al. PatientsLikeMe® Online Epilepsy Community: Patient characteristics and predictors of poor health-related quality of life. Epilepsy & Behavior. 2016;63:20-8.
79. Wicks P, Keininger DL, Massagli MP, de la Loge C, Brownstein C, Isojarvi J, et al. Perceived benefits of sharing health data between people with epilepsy on an online platform. Epilepsy & Behavior. 2012;23(1):16-23.
80. Wicks P, Massagli M, Frost J, Brownstein C, Okun S, Vaughan T, et al. Sharing health data for better outcomes on PatientsLikeMe. Journal of Medical Internet Research. 2010;12(2):e19-e.
81. Smith CA, Wicks PJ. PatientsLikeMe: Consumer health vocabulary as a folksonomy. AMIA Annual Symposium proceedings. 2008;2008:682-6.
82. Sahama T, Liang J, Iannella R. Impact of the social networking applications for health information management for patients and physicians. Studies in health technology and informatics. 2012;180:803-7.
83. Frost JH, Massagli MP, Wicks P, Heywood J. How the Social Web Supports patient experimentation with a new therapy: The demand for patient-controlled and patient-centered informatics. AMIA Annual Symposium proceedings. 2008;2008:217-21.
84. Griffiths F, Dobermann T, Cave JAK, Thorogood M, Johnson S, Salamatian K, et al. The Impact of Online Social Networks on Health and Health Systems: A Scoping Review and Case Studies. Policy and internet. 2015;7(4):473-96.
85. Brownstein CA, Wicks P. The potential research impact of patient reported outcomes on osteogenesis imperfecta. Clin Orthop Relat Res. 2010;468(10):2581-5.
86. Wicks P, Heywood JA. Data donation could power the learning health care system, including special access programs. Am J Bioeth. 2014;14(11):27-9.
87. Okun S, Goodwin K. Building a learning health community: By the people, for the people. Learning health systems. 2017;1(3):e10028-n/a.
88. Chiauzzi E, Wicks P. Digital Trespass: Ethical and Terms-of-Use Violations by Researchers Accessing Data From an Online Patient Community. J Med Internet Res. 2019;21(2):e11985.
89. Trevena LJ. PatientsLikeMe and the tale of three brothers. Medical journal of Australia. 2011;195(5):258-9.
90. Ellis L, Showell C, Turner P. Social media and patient self-management: not all sites are created equal. Studies in health technology and informatics. 2013;183:291-5.
91. Li J. Privacy policies for health social networking sites. Journal of the American Medical Informatics Association : JAMIA. 2013;20(4):704-7.
92. Rundle CW, Dellavalle RP. PatientsLikeMe and atopic dermatitis: characterizing the atopic dermatitis patient profile. Dermatology online journal. 2018;24(8).
93. Wicks P. Patient, study thyself. BMC medicine. 2018;16(1):217-.
94. Wicks P, Sulham KA, Gnanasakthy A. Quality of Life in Organ Transplant Recipients Participating in an Online Transplant Community. The patient : patient-centered outcomes research. 2013;7(1):73-84.
95. O'Brien EC, Rodriguez AM, Kum H-C, Schanberg LE, Fitz-Randolph M, O'Brien SM, et al. Patient perspectives on the linkage of health data for research: Insights

For peer review only

from an online patient community questionnaire. International journal of medical informatics (Shannon, Ireland). 2019;127:9-17.
96. Janssen S, Soneji M, Nonnekes J, Bloem BR. A painted staircase illusion to alleviate freezing of gait in Parkinson's disease. J Neurol. 2016;263(8):1661-2.
97. Janssen S, Wezel RJAv, Soneji M, Nonnekes JH, Bloem BR. Response to: staircase climbing is not solely a visual compensation strategy to alleviate freezing of gait in Parkinson's disease. Journal of neurology. 2017;264(1):177-8.
98. Riggare S, Unruh KT. Patients organise and train doctors to provide better care. BMJ. 2015;351:h6318.
99. Vaidyam A, Roux S, Torous J. Patient Innovation in Investigating the Effects of Environmental Pollution in Schizophrenia: Case Report of Digital Phenotyping Beyond Apps. JMIR Ment Health. 2020;7(8):e19778.
100. Torous J, Roux S. Patient-Driven Innovation for Mobile Mental Health Technology: Case Report of Symptom Tracking in Schizophrenia. JMIR Ment Health. 2017;4(3):e27.
101. Braune K, O'Donnell S, Cleal B, Lewis DM, Tappe A, Hauck B, et al. 117-LB: DIWHY: Factors Influencing Motivation, Barriers, and Duration of DIY Artificial Pancreas System Use among Real-World Users. Diabetes (New York, NY). 2019;68(Supplement_1).
102. Cleal B, Braune K, O'Donnell S, Hauck B, Lewis DM, Skinner TC, et al. 78-LB: Detailing the Experiences of People with Diabetes Using Do-It-Yourself Artificial Pancreas Systems—Qualitative Analysis of Responses to Open-Ended Items in an International Survey. Diabetes (New York, NY). 2019;68(Supplement_1).
103. Hussain S, Lewis D. Diabetes Podcast: Do It Yourself/Open Source Artificial Pancreas Systems: Part 1. Diabetes Therapy. 2020;11(8):1609-19.
104. Hussain S, Lewis D. Diabetes Podcast: Do It Yourself/Open Source Artificial Pancreas Systems: Part 2. Diabetes Therapy. 2020;11(8):1621-6.
105. Lewis DM, Swain RS, Donner TW. Improvements in A1C and Time-in-Range in DIY Closed-Loop (OpenAPS) Users. Diabetes (New York, NY). 2018;67(Supplement 1):352.
106. Zabinsky J, Howell H, Ghezavati A, Lewis DM, Nguyen A, Wong JC. 988-P: Do-It-Yourself Artificial Pancreas Systems for Type 1 Diabetes Reduce Hyperglycemia without Increasing Hypoglycemia. Diabetes. 2020;69(Supplement 1):988-P.
107. Melmer A, Züger T, Lewis D, Leibrand SM, Laimer M. 76-OR: In-Depth Review of Glycemic Control and Glycemic Variability in People with Type 1 Diabetes Using Open Source Artificial Pancreas Systems. Diabetes2019.
108. Heywood J, Zeliadt N. Straight talk with...Jamie Heywood. Nat Med. 2014;20(5):457.
109. Wicks P, Vaughan T, Heywood J. Subjects no more: what happens when trial participants realize they hold the power? BMJ. 2014;348(jan28 9):g368-g.
110. Okun S, Wicks P. DigitalMe: a journey towards personalized health and thriving. Biomedical engineering online. 2018;17(1):119-.
111. Wicks P. Could digital patient communities be the launch pad for patient-centric trial design? Trials. 2014;15.
112. Pearson JF, Brownstein CA, Brownstein JS. Potential for electronic health records and online social networking to redefine medical research. Clinical chemistry (Baltimore, Md). 2011;57(2):196-204.
113. Schroeder EB, Desai J, Schmittdiel JA, Paolino AR, Schneider JL, Goodrich GK, et al. An Innovative Approach to Informing Research: Gathering Perspectives on Diabetes Care Challenges From an Online Patient Community. Interactive journal of medical research. 2015;4(2):e13-e.

For peer review only

114. O'Hair D, Villagran MM, Wittenberg E, Brown K, Ferguson M, Hall HT, et al. Cancer Survivorship and Agency Model: Implications for Patient Choice, Decision Making, and Influence. Health Communication. 2003;15(2):193-202.
115. Canhão H, Zejnilovic L, Oliveira P. Revolutionizing healthcare by empowering patients to innovate. EMJ Innovations. 2017;1[1]:31-34.
116. Oliveira P, Zejnilović L, Canhão H. Challenges and Opportunities in Developing and Sharing Solutions by Patients and Caregivers: The Story of a Knowledge Commons for the Patient Innovation Project. Cambridge University Press; 2017. p. 301-25.
117. Malterud K, Siersma VD, Guassora AD. Sample Size in Qualitative Interview Studies: Guided by Information Power. Qualitative health research. 2016;26(13):1753-60.
118. Demonaco H, Oliveira P, Torrance A, Von Hippel C, Von Hippel E. When patients become innovators. Springer Fachmedien Wiesbaden; 2020. p. 121-9.
119. Zejnilović L, Oliveira P, Canhão H. Innovations by and for Patients, and Their Place in the Future Health Care System. In: Albach H, Meffert H, Pinkwart A, Reichwald R, von Eiff W, editors. Boundaryless Hospital: Rethink and Redefine Health Care Management. Berlin, Heidelberg: Springer Berlin Heidelberg; 2016. p. 341-57.
120. Bradonjic P, Franke N, Lüthje C. Decision-makers' underestimation of user innovation. Research policy. 2019;48(6):1354-61.
121. Nielsen KR. Policymakers' views on sustainable end-user innovation: Implications for sustainable innovation. Journal of cleaner production. 2020;254:120030.
122. Trischler J, Svensson PO, Williams H, Wikström F. Citizens as an innovation source in sustainability transitions - linking the directionality of innovations with the locus of the problem in transformative innovation policy. Public management review. 2022;ahead-of-print(ahead-of-print):1-23.
123. Frow P, McColl-Kennedy JR, Payne A. Co-creation practices: Their role in shaping a health care ecosystem. Industrial Marketing Management. 2016;56:24-39.
124. Halvorsrud K, Kucharska J, Adlington K, Rüdell K, Brown Hajdukova E, Nazroo J, et al. Identifying evidence of effectiveness in the co-creation of research: a systematic review and meta-analysis of the international healthcare literature. Journal of Public Health. 2021;43(1):197-208.
125. von Hippel E, Kaulartz S. Next-generation consumer innovation search: Identifying early-stage need-solution pairs on the web. Research policy. 2021;50(8):104056.

Figure 1: Number and proportion of publications represented in the three inductively created categories representing unmet needs addressed by patient-driven innovations. NB: some publications were included in more than one category.

Figure 2. Number and proportion of publications in the two inductively created categories representing outcomes of patient-driven innovations. NB: in total 65 (78%) of the publications did not report any outcomes.

For peer review only

For peer review only

For peer review only

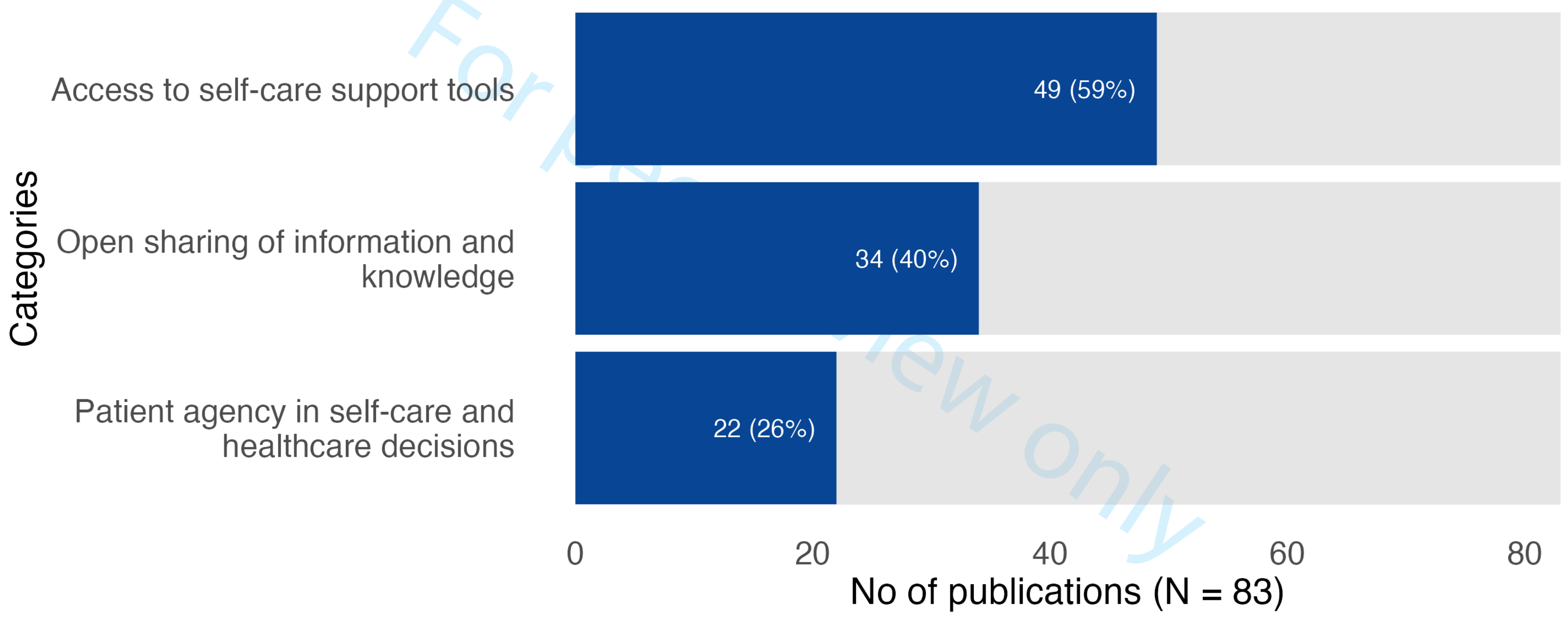

For peer review only

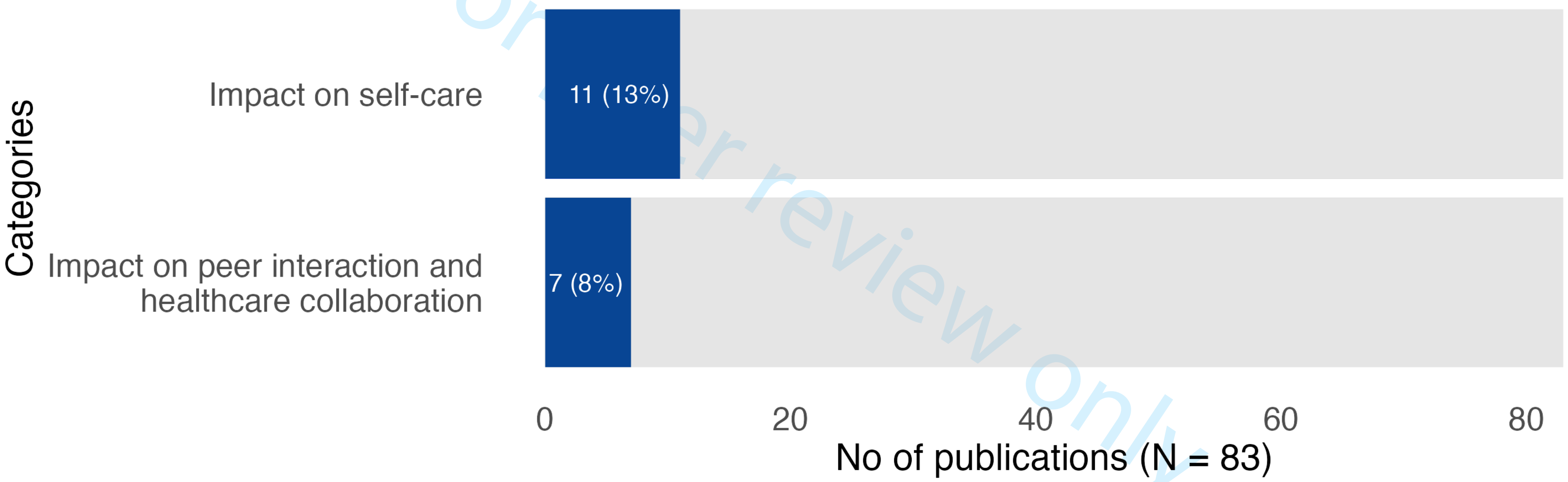

For peer review only

Appendix 1. Description of the patient-driven innovations included in the study, grouped by the medical conditions they addressed.

**Medical condition**	**Innovation name**	**Innovation description**	**References**
22q11 Deletion syndrome	22q11 Ireland	Collaborative network of patients, informal caregivers, clinicians, and researchers for the development a multidisciplinary care coordination and pathways for patients with rare diseases	Lawlor A, Kerin L, Orr D, Leahy R, Crotty F, Kelleher S, et al. Developing integrated care in the context of rare chromosomal conditions: 22q11 Deletion Syndrome; A parent/clinician collaboration. International journal of integrated care. 2017;17(5):215.
Breast cancer	Jacki Jacket	Patient clothing after breast cancer surgery to secure and hide drainage tubes	Berry DL, Blonquist TM, Halpenny B, Hong F, Morrison-Ma SC, McCullough MC, et al. The Jacki Jacket after mastectomy with reconstruction: a randomized pilot study. Breast cancer research and treatment. 2019;179(2):377-85.
Cerebral palsy & hypertonia	Auditory stimulation*	Auditory stimulation to improve motor function in children with cerebral palsy and hypertonia	Ben-Pazi H, Aran A, Pandyan A, Gelkop N, Ginsberg G, Pollak Y, et al. Auditory stimulation improves motor function and caretaker burden in children with cerebral palsy- A randomized double blind study. PLoS One. 2018;13(12):e0208792.
	Upsee	Wearable device. A harness to support gait training for postural control and motor function for children with cerebral palsy and hypertonia	Ardolino E, Flores M, Manella K. Gross Motor Outcomes After Dynamic Weight-Bearing in 2 Children With Trunk Hypotonia: A Case Series. Pediatr Phys Ther. 2017;29(4):360-4.<br><br>Fergus A. A Novel Mobility Device to Improve Walking for a Child With Cerebral Palsy. Pediatr Phys Ther. 2017;29(4):E1-E7.
Cystic fibrosis (CF)	Sweden CF Coalition	Collaborative learning network enabling “persons with CF, families, clinicians, researchers and others to work together toward common goals”	de Monestrol I, Ericson P, Hjelte L, Mostrom J, Hager A, Lindblad S, editors. Co-Creating the Swedish example for orderly introduction and follow-up on new therapies 2018 2018.<br><br>Lindblad A, Hedborg A, Elidottir H, de Monestrol I, Hjelte L, Ericson P, et al., editors. Sweden’s learning health system approach to new therapies: nine months with Lumacaftor/Ivacaftor2019 2019.

For peer review only

Diabetes	Autosense	Technical innovation for diabetes care: A development/version of Do-It-Yourself Artificial Pancreas System (DIY-APS), reviewing and assessing insulin sensitivity and adjust ISF deviations	Lewis DM, Leibrand S, Street TJ, Phatak SS. Detecting Insulin Sensitivity Changes for Individuals with Type 1 Diabetes. Diabetes. 2018;67(Supplement_1).
	Autotune	Technical innovation for diabetes care: a development/version of DIY-APS, builds on Nightscout (below) data and recommends changes to basal rate, insulin/carb ratio and ISF	Lewis D. Automatic Estimation of Basals, ISF, and Carb Ratio for Sensor-Augmented Pump and Hybrid Closed-Loop Therapy. American Diabetes Association's 77th Scientific Sessions San Diego, CA2017.
	DIY-APS, Do-It-Yourself Artificial Pancreas System	Digital technical innovation for diabetes care: a closed loop system that enables access to real-time continuous glucose monitoring (CGM) and automated insulin delivery by connecting CGM technology via a communication device to an insulin pump that operates based on an algorithm calculating insulin doses	Barnard KD, Ziegler R, Klonoff DC, Braune K, Petersen B, Rendschmidt T, et al. Open Source Closed-Loop Insulin Delivery Systems: A Clash of Cultures or Merging of Diverse Approaches? Journal of Diabetes Science & Technology. 2018;12(6):1223-6.<br><br>Bock M. The 'do it yourself' type 1 diabetes dilemma for medical practitioners. Internal Medicine Journal. 2019;49(5):559-61.<br><br>Braune K, O'Donnell S, Cleal B, Lewis D, Tappe A, Willaing I, et al. Real-World Use of Do-It-Yourself Artificial Pancreas Systems in Children and Adolescents With Type 1 Diabetes: Online Survey and Analysis of Self-Reported Clinical Outcomes. JMIR mHealth and uHealth. 2019;7(7):e14087-e.<br><br>Burnside M, Crocket H, Mayo M, Pickering J, Tappe A, de Bock M. Do-It-Yourself Automated Insulin Delivery: A Leading Example of the Democratization of Medicine. Journal of diabetes science and technology. 2020;14(5):878-82.<br><br>Crabtree TSJ, McLay A, Wilmot EG. DIY artificial pancreas systems: here to stay? Practical Diabetes. 2019;36(2):63-8.<br><br>Dowling L, Wilmot EG, Choudhary P. Do-it-yourself closed-loop systems for people living with type 1 diabetes. Diabetic Medicine. 2020;37(12):1977-80.

For peer review only

		Farrington C. Hacking diabetes: DIY artificial pancreas systems. Lancet Diabetes Endocrinol. 2017;5(5):332.<br><br>Hng TM, Burren D. Appearance of Do-It-Yourself closed-loop systems to manage type 1 diabetes. Intern Med J. 2018;48(11):1400-4.<br><br>Jennings P, Hussain S. Do-It-Yourself Artificial Pancreas Systems: A Review of the Emerging Evidence and Insights for Healthcare Professionals. J Diabetes Sci Technol. 2020;14(5):868-77.<br><br>Lee JM, Hirschfeld E, Wedding J. A Patient-Designed Do-It-Yourself Mobile Technology System for Diabetes: Promise and Challenges for a New Era in Medicine. JAMA. 2016;315(14):1447-8.<br><br>Lemieux P, Yamamoto J, Donovan L. 44 - Do-It-Yourself Artificial Pancreas System Use in Pregnancy in a Real-World Setting: A Case Report. Canadian Journal of Diabetes. 2020;44(7):S20.<br><br>Lewis D, Leibrand S, Open APSC. Real-World Use of Open Source Artificial Pancreas Systems. J Diabetes Sci Technol. 2016;10(6):1411.<br><br>Lewis D. History and Perspective on DIY Closed Looping. J Diabetes Sci Technol. 2019;13(4):790-3.<br><br>Lewis D. Setting Expectations for Successful Artificial Pancreas/Hybrid Closed Loop/Automated Insulin Delivery Adoption. J Diabetes Sci Technol. 2018;12(2):533-4.<br><br>Lewis DM, Grant AD. 1056-P: Characterization of Multi-timescale Biological Rhythms in Individuals with Type 1 Diabetes. Diabetes. 2019;68 (Supplement_1).<br><br>Lewis DM, Leibrand S. How a DIY Artificial Pancreas Built by a Patient Makes New Tools Available for Clinicians. American Diabetes Association Scientific Sessions 2015.

For peer review only

		Litchman ML, Lewis D, Kelly LA, Gee PM. Twitter Analysis of #OpenAPS DIY Artificial Pancreas Technology Use Suggests Improved A1C and Quality of Life. Journal of diabetes science and technology. 2019;13(2):164-70.<br>Litchman ML, Walker HR, Fitzgerald C, Gomez Hoyos M, Lewis D, Gee PM. Patient-Driven Diabetes Technologies: Sentiment and Personas of the #WeAreNotWaiting and #OpenAPS Movements. Journal of Diabetes Science and Technology. 2020;14(6):990-9.<br><br>Marshall DC, Holloway M, Korer M, Woodman J, Brackenridge A, Hussain S. Do-It-Yourself Artificial Pancreas Systems in Type 1 Diabetes: Perspectives of Two Adult Users, a Caregiver and Three Physicians. Diabetes Ther. 2019;10(5):1553-64.<br><br>Melmer A, Zger T, Lewis DM, Leibrand S, Stettler C, Laimer M. Glycaemic control in individuals with type 1 diabetes using an open source artificial pancreas system (OpenAPS). Diabetes Obesity & Metabolism. 2019;21(10):2333-7.<br><br>Murray JA, Clayton MF, Litchman ML. Health Care Provider Knowledge and Perceptions of FDA-Approved and Do-It-Yourself Automated Insulin Delivery. Journal of diabetes science and technology. 2020;14(6):1017-21.<br><br>O'Donnell S, Lewis D, Marchante Fernández M, Wäldchen M, Cleal B, Skinner T, et al. Evidence on User-Led Innovation in Diabetes Technology (The OPEN Project): Protocol for a Mixed Methods Study. JMIR Research Protocols. 2019;8(11):e15368.<br><br>Oliver N, Reddy M, Marriott C, Walker T, Heinemann L. Open source automated insulin delivery: addressing the challenge. NPJ digital medicine. 2019;2(1):124-.<br><br>Omer T. Empowered citizen 'health hackers' who are not waiting. BMC Medicine. 2016;14(1):118.

For peer review only

		Shaw D, Crabtree TSJ, Hammond P, McLay A, Wilmot EG. The DIY artificial pancreas system: an ethical dilemma for doctors. Diabetic medicine. 2020;37(11):1951-3.<br><br>Shepard JA, Breton M, Nimri R, Roberts JTF, Street T, Klonoff D, et al. User and Healthcare Professional Perspectives on Do-It-Yourself Artificial Pancreas Systems: A Need for Guidelines. Journal of diabetes science and technology. 2022;16(1):224-7.
MySugr	Mobile app, stores diabetes data from connected devices, manual entries, and integrations	Debong F, Mayer H, Kober J. Real-World Assessments of mySugr Mobile Health App. Diabetes Technol Ther. 2019;21(S2):S235-S40.<br><br>Mader JK, Sourij H, Laimer M, Debong F, Schandl B, Pieber TR. Influence of frequeant use of mobile health technology on blood glucose control in patientw with type 1 diabetes 8th International Conference onAdvanced Technologies & Treatments for Diabetes February 18–21, 2015; Paris, France2015.
Nightscout	Technical innovation for diabetes care. Allows remote monitoring of glucose levels of Type 1 diabetes using existing monitoring devices	Årsand E, Bradway M, Muzny M, Blixgård H, Grøttland A, Giordanengo A, et al. Warning: the Do-It-Yourself (DIY) wave will drastically change diabetes care! International Journal of Integrated Care. 2016;16(5):2.<br><br>Beckman D, Reehorst CM, Henriksen A, Muzny M, Arsand E, Hartvigsen G. Better glucose regulation through enabling group-based motivational mechanisms in cloud-based solutions like Nightscout. International Journal of Integrated Care. 2016;16.<br><br>Kublin O, Stępień M. The Nightscout system – description of the system and its evaluation in scientific publications. Pediatric Endocrinology Diabetes and Metabolism. 2020;26(3):140-3.<br><br>Lee JM, Newman MW, Gebremariam A, Choi P, Lewis D, Nordgren W, et al. Real-World Use and Self-Reported Health Outcomes of a Patient-Designed Do-it-Yourself Mobile Technology System for Diabetes: Lessons for Mobile Health. Diabetes Technology & Therapeutics. 2017;19(4):209-19.Journal of diabetes science and technology. 2022;16(1):224-7.

For peer review only

		Ng M, Borst E, Garrity A, Hirschfeld E, Lee J. Evolution of Do-It-Yourself Remote Monitoring Technology for Type 1 Diabetes. Journal of Diabetes Science and Technology. 2020;14(5):854-9.<br><br>Rivard L, Lehoux P, Alami H. "It's not just hacking for the sake of it": a qualitative study of health innovators' views on patient-driven open innovations, quality and safety. BMJ Quality & Safety. 2021;30(9):731-8.<br><br>White K, Gebremariam A, Lewis D, Nordgren W, Wedding J, Pasek J, et al. Motivations for Participation in an Online Social Media Community for Diabetes. Journal of Diabetes Science and Technology. 2018;12(3):712-8.
Omnipod	Technical innovation for diabetes care. A wearable tubeless pump for continuous insulin delivery based on temporary basal rates guided by glucose rise and fall	Lebenthal Y, Lazar L, Benzaquen H, Shalitin S, Phillip M. Patient perceptions of using the OmniPod system compared with conventional insulin pumps in young adults with type 1 diabetes. Diabetes Technol Ther. 2012;14(5):411-7.<br><br>Zisser H, Breton M, Dassau E, Markova K, Bevier W, Seborg D, et al. Novel Methodology to Determine the Accuracy of the OmniPod Insulin Pump: A Key Component of the Artificial Pancreas System. Journal of Diabetes Science and Technology. 2011;5(6):1509-18.
T1resources.uk	Online network. Co-created resource library by healthcare professionals and persons living with Type1 Diabetes to collect and offer valuable and trustworthy self-care resources for diabetes patients	Kendall M, Cleverly L, Winchcombe K, Kar P, Choudhary P, Harris S. T1resources.uk; rated, reviewed, reliable. A co-created website for people affected by type 1 diabetes. British Journal of Diabetes. 2017;17(3).
Webdia	Mobile app for children with Type 1 Diabetes to improve diabetes control by calculating insulin dose and information about meals	Klee P, Bussien C, Castellsague M, Combescure C, Dirlewanger M, Girardin C, et al. An Intervention by a Patient-Designed Do-It-Yourself Mobile Device App Reduces HbA1c in Children and Adolescents with Type 1 Diabetes: A Randomized Double-Crossover Study. Diabetes Technol Ther. 2018;20(12):797-805.

For peer review only

Gastrointestinal diseases	Helminth therapy*	Alternative treatment. Experimental treatment of Crohn's disease by ingesting pig whipworm	Ahrens S. Opening (and Swallowing) A Can of Worms to Treat My Crohn's Disease. Am J Gastroenterol. 2016;111(7):918-20.
	Ostom-i-Alert	Wearable device. An app consisting of a Bluetooth linked stoma bag biosensor that records and alerts patients in real-time to prevent pouch spill	Kontovounisios C, Smith J, Dawson P, Warren O, Mills S, Von Roon A, et al. The Ostom-i Alert Sensor: a new device to measure stoma output. Tech Coloproctol. 2018;22(9):697-701.<br><br>Rouholiman D, Gamble JG, Dobrota SD, Encisco EM, Shah AG, Grajales Iii FJ, et al. Improving Health-Related Quality of Life of Patients With an Ostomy Using a Novel Digital Wearable Device: Protocol for a Pilot Study. JMIR Res Protoc. 2018;7(3):e82.<br><br>Seres M. From Patient to Patient-Entrepreneur: Development of an Ostomy Bag Sensor. Am J Gastroenterol. 2018;113(1):8-10.
Multiple conditions	Genia	App-based patient support system to foster collaborative care to facilitate transition from pediatric to adult care among young chronically ill patients	Grande SW, Longacre MR, Palmblad K, Montan MV, Berquist RP, Hager A, et al. Empowering Young People Living With Juvenile Idiopathic Arthritis to Better Communicate With Families and Care Teams: Content Analysis of Semistructured Interviews. JMIR mHealth and uHealth. 2019;7(2):e10401-e.<br><br>Longacre M, Grande S, Hager A, Montan M, Bergquist RP, Martensson M, et al. Clinical Adoption of mHealth Technology to Support Pediatric Cystic Fibrosis Care in Sweden: Qualitative Case Study. JMIR Pediatr Parent. 2018;1(2):e11080
	MediStori	A paper based personal health record and a self-management toolkit for families	O'Connor O. The MediStori. A personal health record and standardised self-management toolkit which can improve integrated care systems. International Journal of Integrated Care. 2017;17:1-5.
	PatientsLikeMe	Online network empowering patients by enabling sharing of longitudinal health data and information on illness experience (symptoms and treatment and experiences of self-care management through visible profiles	Ma X, Sayama H. Mental disorder recovery correlated with centralities and interactions on an online social network. PeerJ (San Francisco, CA). 2015;3:e1163-e.<br><br>Brownstein CA, Brownstein JS, Williams DS, 3rd, Wicks P, Heywood JA. The power of social networking in medicine. Nat Biotechnol. 2009;27(10):888-90.

For peer review only

		Williams III DS. The PatientsLikeMe® Multiple Sclerosis Community: Using online marketing to shift the health data privacy paradigm. Journal of communication in healthcare. 2010;3(1):48-61.<br><br>Frost J, Massagli M. PatientsLikeMe the case for a data-centered patient community and how ALS patients use the community to inform treatment decisions and manage pulmonary health. Chronic Respiratory Disease. 2009;6(4):225-9.<br><br>Frost JH, Massagli MP. Social uses of personal health information within PatientsLikeMe, an online patient community: what can happen when patients have access to one another's data. J Med Internet Res. 2008;10(3):e15.<br><br>De La Loge C, Dimova S, Mueller K, Phillips G, Durgin TL, Wicks P, et al. PatientsLikeMe® Online Epilepsy Community: Patient characteristics and predictors of poor health-related quality of life. Epilepsy & Behavior. 2016;63:20-8.<br><br>Wicks P, Keininger DL, Massagli MP, de la Loge C, Brownstein C, Isojarvi J, et al. Perceived benefits of sharing health data between people with epilepsy on an online platform. Epilepsy & Behavior. 2012;23(1):16-23.<br><br>Wicks P, Massagli M, Frost J, Brownstein C, Okun S, Vaughan T, et al. Sharing health data for better outcomes on PatientsLikeMe. Journal of Medical Internet Research. 2010;12(2):e19-e.<br><br>Smith CA, Wicks PJ. PatientsLikeMe: Consumer health vocabulary as a folksonomy. AMIA Annual Symposium proceedings. 2008;2008:682-6.<br><br>Sahama T, Liang J, Iannella R. Impact of the social networking applications for health information management for patients and physicians. Studies in health technology and informatics. 2012;180:803-7.

For peer review only

		Frost JH, Massagli MP, Wicks P, Heywood J. How the Social Web Supports patient experimentation with a new therapy: The demand for patient-controlled and patient-centered informatics. AMIA Annual Symposium proceedings. 2008;2008:217-21.<br><br>Griffiths F, Dobermann T, Cave JAK, Thorogood M, Johnson S, Salamatian K, et al. The Impact of Online Social Networks on Health and Health Systems: A Scoping Review and Case Studies. Policy and internet. 2015;7(4):473-96.<br><br>Brownstein CA, Wicks P. The potential research impact of patient reported outcomes on osteogenesis imperfecta. Clin Orthop Relat Res. 2010;468(10):2581-5.<br><br>Wicks P, Heywood JA. Data donation could power the learning health care system, including special access programs. Am J Bioeth. 2014;14(11):27-9.<br><br>Okun S, Goodwin K. Building a learning health community: By the people, for the people. Learning health systems. 2017;1(3):e10028-n/a.<br><br>Chiauzzi E, Wicks P. Digital Trespass: Ethical and Terms-of-Use Violations by Researchers Accessing Data From an Online Patient Community. J Med Internet Res. 2019;21(2):e11985.<br><br>Trevena LJ. PatientsLikeMe and the tale of three brothers. Medical journal of Australia. 2011;195(5):258-9.<br><br>Ellis L, Showell C, Turner P. Social media and patient self-management: not all sites are created equal. Studies in health technology and informatics. 2013;183:291-5.<br><br>Li J. Privacy policies for health social networking sites. Journal of the American Medical Informatics Association : JAMIA. 2013;20(4):704-7.

For peer review only

			Rundle CW, Dellavalle RP. PatientsLikeMe and atopic dermatitis: characterizing the atopic dermatitis patient profile. Dermatology online journal. 2018;24(8).<br>Wicks P. Patient, study thyself. BMC medicine. 2018;16(1):217-.<br><br>Wicks P, Sulham KA, Gnanasakthy A. Quality of Life in Organ Transplant Recipients Participating in an Online Transplant Community. The patient : patient-centered outcomes research. 2013;7(1):73-84.<br><br>O'Brien EC, Rodriguez AM, Kum H-C, Schanberg LE, Fitz-Randolph M, O'Brien SM, et al. Patient perspectives on the linkage of health data for research: Insights from an online patient community questionnaire. International journal of medical informatics (Shannon, Ireland). 2019;127:9-17.
Parkinson's disease	3D cue illusions*	Optical illusion by painting on the floor allowing externally guided steps for patients with Parkinson's disease to alleviate freezing of gait	Janssen S, Soneji M, Nonnekes J, Bloem BR. A painted staircase illusion to alleviate freezing of gait in Parkinson's disease. J Neurol. 2016;263(8):1661-2.<br><br>Janssen S, Wezel RJAv, Soneji M, Nonnekes JH, Bloem BR. Response to: staircase climbing is not solely a visual compensation strategy to alleviate freezing of gait in Parkinson's disease. Journal of neurology. 2017;264(1):177-8.
	Prototype of a mobile app for self-care*	Prototype of a mobile app developed by lead user patients in Parkinson's disease, collecting data on drug intake and well-being for shared decision-making with neurologist	Riggare S, Unruh KT. Patients organise and train doctors to provide better care. BMJ. 2015;351:h6318.
Schizophrenia	Hallucination tracking*	Technical innovation for self-tracking of auditory hallucinations in schizophrenia patients	Torous J, Roux S. Patient-Driven Innovation for Mobile Mental Health Technology: Case Report of Symptom Tracking in Schizophrenia. JMIR Ment Health. 2017;4(3):e27.<br><br>Vaidyam A, Roux S, Torous J. Patient Innovation in Investigating the Effects of Environmental Pollution in Schizophrenia: Case Report of Digital Phenotyping Beyond Apps. JMIR Ment Health. 2020;7(8):e19778.

For peer review only

* A descriptive label is presented for innovations without a name.

For peer review only

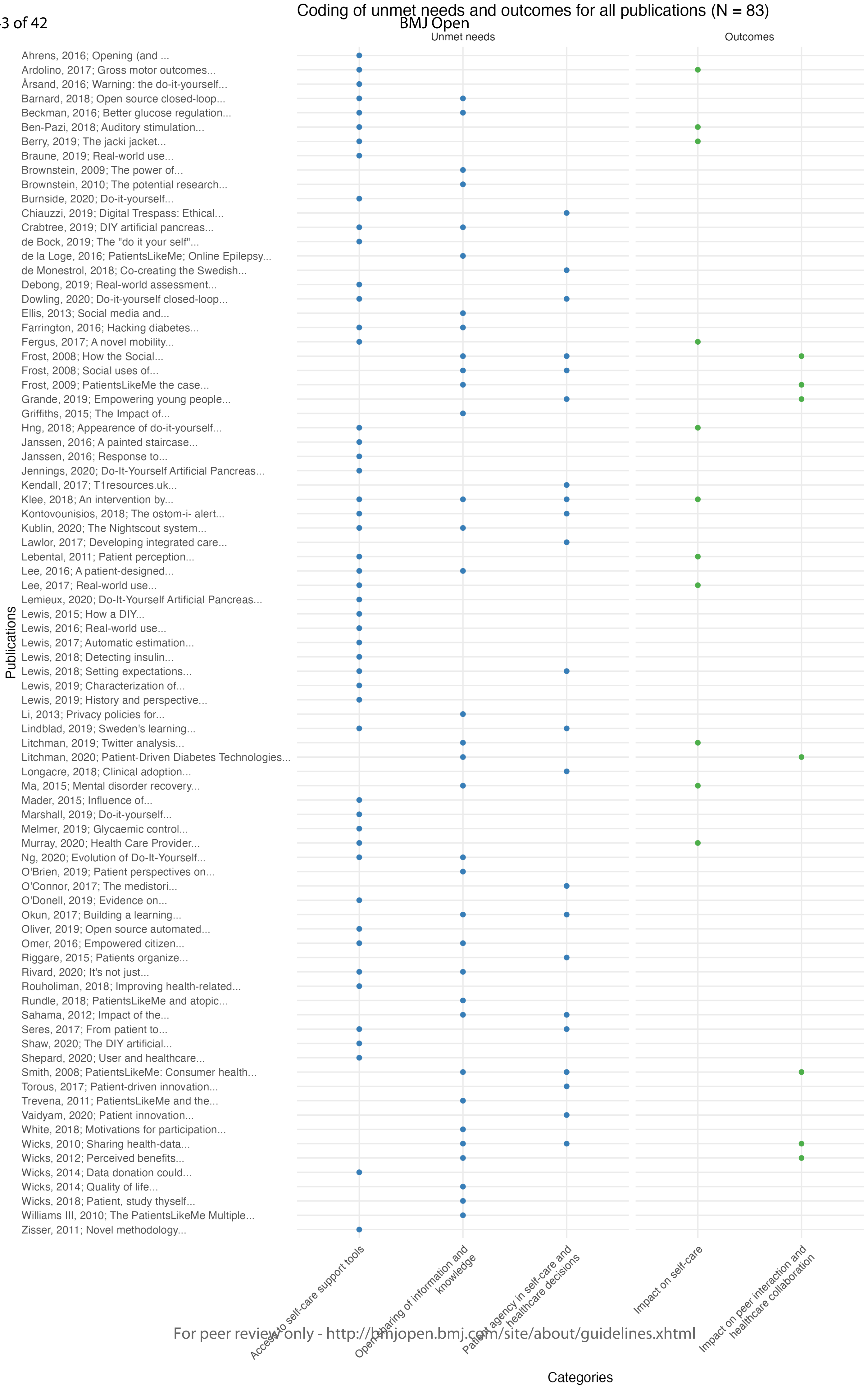
Coding of unmet needs and outcomes for all publications (N = 83)
Unmet needs
Outcomes
Ahrens, 2016; Opening (and ...
Ardolino, 2017; Gross motor outcomes...
Årsand, 2016; Warning: the do-it-yourself...
Barnard, 2018; Open source closed-loop...
Beckman, 2016; Better glucose regulation...
Ben-Pazi, 2018; Auditory stimulation...
Berry, 2019; The jacki jacket...
Braune, 2019; Real-world use...
Brownstein, 2009; The power of...
Brownstein, 2010; The potential research...
Burnside, 2020; Do-it-yourself...
Chiauzzi, 2019; Digital Trespass: Ethical...
Crabtree, 2019; DIY artificial pancreas...
de Bock, 2019; The "do it your self"...
de la Loge, 2016; PatientsLikeMe; Online Epilepsy...
de Monestrol, 2018; Co-creating the Swedish...
Debong, 2019; Real-world assessment...
Dowling, 2020; Do-it-yourself closed-loop...
Ellis, 2013; Social media and...
Farrington, 2016; Hacking diabetes...
Fergus, 2017; A novel mobility...
Frost, 2008; How the Social...
Frost, 2008; Social uses of...
Frost, 2009; PatientsLikeMe the case...
Grande, 2019; Empowering young people...
Griffiths, 2015; The Impact of...
Hng, 2018; Appearence of do-it-yourself...
Janssen, 2016; A painted staircase...
Janssen, 2016; Response to...
Jennings, 2020; Do-It-Yourself Artificial Pancreas...
Kendall, 2017; T1resources.uk...
Klee, 2018; An intervention by...
Kontovounisios, 2018; The ostom-i- alert...
Kublin, 2020; The Nightscout system...
Lawlor, 2017; Developing integrated care...
Lebental, 2011; Patient perception...
Lee, 2016; A patient-designed...
Lee, 2017; Real-world use...
Lemieux, 2020; Do-It-Yourself Artificial Pancreas...
Lewis, 2015; How a DIY...
Lewis, 2016; Real-world use...
Lewis, 2017; Automatic estimation...
Lewis, 2018; Detecting insulin...
Lewis, 2018; Setting expectations...
Lewis, 2019; Characterization of...
Lewis, 2019; History and perspective...
Li, 2013; Privacy policies for...
Lindblad, 2019; Sweden's learning...
Litchman, 2019; Twitter analysis...
Litchman, 2020; Patient-Driven Diabetes Technologies...
Longacre, 2018; Clinical adoption...
Ma, 2015; Mental disorder recovery...
Mader, 2015; Influence of...
Marshall, 2019; Do-it-yourself...
Melmer, 2019; Glycaemic control...
Murray, 2020; Health Care Provider...
Ng, 2020; Evolution of Do-It-Yourself...
O'Brien, 2019; Patient perspectives on...
O'Connor, 2017; The medistori...
O'Donell, 2019; Evidence on...
Okun, 2017; Building a learning...
Oliver, 2019; Open source automated...
Omer, 2016; Empowered citizen...
Riggare, 2015; Patients organize...
Rivard, 2020; It's not just...
Rouholiman, 2018; Improving health-related...
Rundle, 2018; PatientsLikeMe and atopic...
Sahama, 2012; Impact of the...
Seres, 2017; From patient to...
Shaw, 2020; The DIY artificial...
Shepard, 2020; User and healthcare...
Smith, 2008; PatientsLikeMe: Consumer health...
Torous, 2017; Patient-driven innovation...
Trevena, 2011; PatientsLikeMe and the...
Vaidyam, 2020; Patient innovation...
White, 2018; Motivations for participation...
Wicks, 2010; Sharing health-data...
Wicks, 2012; Perceived benefits...
Wicks, 2014; Data donation could...
Wicks, 2014; Quality of life...
Wicks, 2018; Patient, study thyself...
Williams III, 2010; The PatientsLikeMe Multiple...
Zisser, 2011; Novel methodology...
Publications
Access to self-care support tools
Open sharing of information and knowledge
Patient agency in self-care and healthcare decisions
Impact on self-care
Impact on peer interaction and healthcare collaboration
Categories
